# Supplementary material for: The Carnivoran Adaptive Landscape Reveals Trade-offs among Functional Traits in the Skull, Appendicular, and Axial Skeleton
Source: Integr Org Biol. 2025 Jan 11;7(1):obaf001. doi: 10.1093/iob/obaf001 (PMC11756339; doi:10.1093/iob/obaf001)
Supplement: obaf001_Supplemental_File [file obaf001_supplemental_file.pdf]

Supplementary Materials for

The carnivoran adaptive landscape reveals functional trade-offs among the skull, appendicular, and axial skeleton

Chris J. Law[1,2], Leslea J. Hlusko[3], Z. Jack Tseng[2]

[1]Burke Museum and Department of Biology, University of Washington

[2]Department of Integrative Biology, University of California Berkeley

[3]National Research Center on Human Evolution (CENIEH), Burgos, Spain

Correspondence: cjlaw@uw.edu

Contains:

Supplementary Methods

Fig. S1. Skeletal trait measurements used in this study.

Table S1. PC loadings of skeletal traits.

Table S3. Relative weights of functional proxies optimized in the locomotor landscapes.

Table S4. Pairwise significance tests among dietary adaptive landscapes.

Table S5. Relative weights of functional proxies optimized in the dietary landscapes.

Table S6. Relative weights of functional proxies optimized in the familial landscapes.

Table S7. Pairwise significance tests among familial adaptive landscapes.

Supplementary Results

Fig. S2. Performance surfaces based on theoretical morphology.

Fig. S3. Adaptive landscapes optimized for locomotor groups based on theoretical morphology.

Fig. S4 Adaptive landscapes optimized for dietary groups based on theoretical morphology.

Fig. S5. Adaptive landscapes optimized for families based on theoretical morphology.

Table S8. Relative weights of functional proxies optimized in the locomotor landscapes.

Table S9. Pairwise significance tests among locomotor landscapes based on theoretical morphology.

Table S10. Pairwise significance tests among dietary landscapes based on theoretical morphology.

Table S11. Relative weights of functional proxies optimized in the dietary landscapes based on theoretical morphology.

Table S12. Relative weights of functional proxies optimized in the familial landscapes based on theoretical morphology.

Table S13. Pairwise significance tests among familial adaptive landscapes based on theoretical morphology.

Sensitivity Analyses

Fig. S6. Phylomorphospace of carnivoran skeletal system based on non-size corrected traits.

Fig. S7. Performance surfaces from Sensitivity Analyses 4.

Fig. S8. Adaptive landscapes from Sensitivity Analyses 4.

Table S14. Relative weights of locomotor adaptive landscapes from Sensitivity Analyses 4.

Table S15. Relative weights of dietary adaptive landscapes from Sensitivity Analyses 4.

Table S16. Relative weights of familial adaptive landscapes from Sensitivity Analyses 4.

Fig. S9. Adaptive landscape results from Sensitivity Analyses 2.

Table S17. Scaling matrix for the first linear discriminant function.

Table S18. Relative weights of landscapes from Sensitivity Analyses 2.

References

Supplementary Methods

Creating morphological proxies for function

From these theoretical morphological traits, we calculated 27 functional indices (Table 1) that capture the functional diversity of the skull (Greaves 2012), limbs (Davis 1964; Samuels et al. 2013), and vertebral column (Boszczyk et al. 2001; Jones et al. 2020, 2021):

Functional proxies for the skull

Temporalis mechanical advantage (temMA) = temporalis moment arm / distance from the jaw condyle to the carnassial. temMA estimates how much force is produced at the bite point from force being input by the temporalis muscle. Lever mechanics suggest that a high temMA indicates a jaw optimized for stronger bites whereas a low temMA indicates a jaw optimized for faster bites. Biomechanical studies have found that increased temMA contributes to increased bite force (Smith and Savage 1956; Throckmorton and Dean 1994; Santana et al. 2012; Law et al. 2016).

Masseter mechanical advantage (masMA) = masseter moment arm / distance from the jaw condyle to the carnassial. masMA estimates how much force is produced at the bite point from force being input by the masseter muscle. Lever mechanics suggest that a high masMA indicates a jaw optimized for stronger bites whereas a low masMA indicates a jaw optimized for faster bites. Biomechanical studies have found that increased masMA contributes to increased bite force (Smith and Savage 1956; Throckmorton and Dean 1994; Santana et al. 2012; Law et al. 2016).

Functional proxies for the forelimb

Scapula index (SI) = scapula length / scapula height. SI describes the expansion of shoulder musculature versus contribution of scapula to limb elongation. The absence or reduction of the clavicle in several mammalian clades allows the scapula to move freely in the parasagittal plane and contribute to stride length (Gasc 2001; Polly 2007; Schmidt and Fischer 2009). Some view the scapula as the first element of the forelimb and thus serves as the functional equivalent to the femur in the hindlimb. Biomechanical analyses found that the proximal elements of the limb (i.e., scapula and femur) produce more than half of the propulsive movement of the whole limb at symmetrical gaits (Fischer et al. 2002). Although the relationship between these performance metrics and scapula shape remains to be tested, ecomorphological studies suggest that increased SI helps with increased stride length and thus tends to be associated with cursorial species, whereas decreased SI provides the teres major muscle with more efficient leverage for powerful adduction of the forelimb and thus tends to be associated with fossorial and natatorial species (Polly 2007).

Brachial index (BI) = radius length / humerus length. BI estimates the relative proportions of the proximal and distal elements of the forelimb and serves as an index of the relative distal out-lever length. The relatively longer distal end of the forelimb facilitates increased stride length and decreased moment of inertia of limbs, which in turn decreases the energetic cost of transport (Kram and Taylor 1990; Strang and Steudel 1990; Garland and Janis 1993; Polly 2007; Pontzer 2007a, 2007b; Kilbourne and Hoffman 2013). Performance studies found that increased BI is correlated with faster running speeds (Christiansen 2002). Ecomorphological studies suggest that increased BI tends to be associated with cursorial species whereas decreased BI is associated with arboreal, natatorial, and fossorial species (Davis 1964; Samuels and Valkenburgh 2008; Samuels et al. 2013).

Humeral robustness index (HRI) = mediolateral diameter of humerus / humerus length. HRI estimates the robustness of the humerus and its ability to resist bending and shearing stresses. Performance studies found that increased HRI is correlated with lower stresses at yield, lower elastic moduli, and higher resistance to failure when a bending force is applied (Kemp et al. 2005). Ecomorphological studies suggest that increased HRI tends to be associated with fossorial and natatorial species whereas decreased HRI is associated with cursorial and scansorial species (Samuels and Valkenburgh 2008; Samuels et al. 2013; Kilbourne 2017; Rickman et al. 2023).

Humeral epicondylar index (HEI) = epicondylar breadth of humerus / humerus length. HEI estimates the relative area of the distal end of the humerus available for the origins of the forearm flexors, pronators, and supinators. Ecomorphological studies suggest that increased HEI tends to be associated with increased fossorial behaviors by helping generate power, force, and stability (Davis 1964; Hildebrand 1985; Lessa and Stein 1992; Lagaria and Youlatos 2006; Samuels and Valkenburgh 2008; Samuels et al. 2013; Rose et al. 2014; Rickman et al. 2023). However, the relationship between HEI and sizes of the forearm flexors, pronators, and supinators remains largely untested.

Olecranon length index (OLI) = olecranon process length / ulna length. OLI estimates the relative mechanical advantage of the triceps brachii and dorsoepitrochlearis muscles used in elbow extension. Biomechanical calculations indicates that an enlarged olecranon process facilitates stronger extension and flexion of the elbow and wrist by increasing mechanical advantage of these muscles, which in turn facilitate the ability to generate large forces (Smith and Savage 1956). Ecomorphological studies suggest that increased OLI is associated with increased fossoriality (Davis 1964; Hildebrand 1985; Lessa and Stein 1992; Lagaria and

Youlatos 2006; Samuels and Valkenburgh 2008; Samuels et al. 2013; Rose et al. 2014; Rickman et al. 2023).

Ulnar robustness index (URI) = mediolateral diameter of ulna / ulna length. URI estimates the robustness of the ulna and its ability to resist bending and shearing stresses, and relative area available for the origin and insertion of forearm and manus flexors, pronators, and supinators. Performance studies on URI are limited. URI is presumably similar to radial robustness, where increased radial robustness is correlated with lower stresses at yield, lower elastic moduli, and higher resistance to failure when a bending force is applied (Kemp et al. 2005). Ecomorphological studies suggest that increased URI tends to be associated with fossorial and natatorial species whereas decreased URI tends to be associated with cursorial and scansorial species (Samuels and Valkenburgh 2008; Samuels et al. 2013; Kilbourne 2017; Rickman et al. 2023).

Manus proportions index (MAN) = metacarpal 3 length / humerus length. MAN estimates the relative proportions of proximal and distal elements of the forelimb, and relative size of the hand. Performance studies found that increased MAN is correlated with faster running speeds (Christiansen 2002).

Functional proxies for the hindlimb

Crural index (CI) = tibia length / femur length. CI estimates relative proportions of proximal and distal elements of the hind limb. The relative longer distal end of the hindlimb facilitate increased stride length and decreased moment of inertia of limbs, which in turn decreases the energetic cost of transport (Kram and Taylor 1990; Strang and Steudel 1990; Garland and Janis 1993; Polly 2007; Pontzer 2007a, 2007b; Kilbourne and Hoffman 2013).

Performance studies found that increased CI is correlated with faster running speeds (Christiansen 2002). Ecomorphological studies suggest that increased CI tends to be associated with cursorial species whereas decreased CI tends to be associated with arboreal, natatorial, and fossorial species and (Davis 1964; Samuels and Valkenburgh 2008; Samuels et al. 2013).

Femoral robustness index (FRI) = anteroposterior diameter of femur / femur length. Estimates robustness of the femur and its ability to resist bending and shearing stresses. Performance studies found that increased FRI is correlated with lower stresses at yield, lower elastic moduli, and higher resistance to failure when a bending force is applied (Kemp et al. 2005). Ecomorphological studies suggest increased FRI tends to be associated with fossorial and natatorial species whereas decreased FRI tends to be associated with cursorial and scansorial species (Samuels and Valkenburgh 2008; Samuels et al. 2013; Kilbourne 2017; Rickman et al. 2023).

Gluteal index (GI) = length of distal extension of the greater trochanter of the femur / femur length. GI estimates the relative mechanical advantage of the gluteal muscles used in retraction of the femur. Ecomorphological studies suggest increased GI tends to be associated with fossorial (Samuels and Valkenburgh 2008). However, empirical performance data investigating the functional advantages of increased GI remains largely untested.

Femoral epicondylar index (FEI) = epicondylar breadth of femur / femur length. FEI estimates the relative area available for the origins of the gastrocnemius and soleus muscles used in extension of the knee and plantar-flexion of the pes. Ecomorphological studies suggest that increased FEI tends to be associated with increased fossorial behaviors by helping generate power, force, and stability (Davis 1964; Hildebrand 1985; Lessa and Stein 1992; Lagaria and Youlatos 2006; Samuels and Valkenburgh 2008; Samuels et al. 2013; Rose et al. 2014; Rickman

et al. 2023). However, empirical performance data investigating the relationship between FEI and sizes of the gastrocnemius and soleus muscles remains largely untested.

Tibial robustness index (TRI) = mediolateral diameter of tibia / tibia length. TRI estimates robustness of the tibia and its ability to resist bending and shearing stresses. Performance studies found that increased TRI is correlated with lower stresses at yield, lower elastic moduli, and higher resistance to failure when a bending force is applied (Kemp et al. 2005). Ecomorphological studies suggest increased TRI tends to be associated with fossorial and natatorial species whereas decreased TRI tends to be associated with cursorial and scansorial species (Samuels and Valkenburgh 2008; Samuels et al. 2013; Kilbourne 2017; Rickman et al. 2023).

Pes length index (PES) = metatarsal 3 length / femur length. Estimates relative proportions of proximal and distal elements of the hind limb, and relative size of the hind foot. Performance studies found that increased PES is correlated with faster running speeds (Garland and Janis 1993; Christiansen 2002).

Functional proxies for the vertebrae

Because the carnivoran vertebral column can be divided into three regionalized modules (i.e., cervical, pre-diaphragmatic thoracic, and post-diaphragmatic thoracolumbar) with the diaphragmatic vertebra serving as a transitional vertebra (Martín-Serra et al. 2021), we only included the functional proxies of the fifth cervical vertebra, middle thoracic vertebra, diaphragmatic vertebra, and middle lumbar vertebra as our proxies for these four regions.

Sagittal second moment of area (sSMA) = $\pi/4$ * breadth of posterior centrum * height of the posterior centrum^3. sSMA estimates the stiffness in the of the vertebral joint in the sagittal

plane. Biomechanical testing using cadaveric bending experiments found a strong correlation (r = 0.95) between sSMA and compliance (inverse of stiffness) (Jones et al. 2020).

Lateral second moment of area (lSMA) = $\pi/4$ * height of posterior centrum * breadth of the posterior centrum^3. lSMA estimates the stiffness in the of the vertebral joint in the lateral plane. Biomechanical testing using cadaveric bending experiments found a strong correlation (r = 0.95) between sSMA and compliance (inverse of stiffness) (Jones et al. 2020).

Joint torsional angle (JTA) = 360º – absolute value of 270º – angle between postzygapophyses. JTA estimates the degree of axial torsion of the vertebrae. Biomechanical testing using cadaveric bending experiments found a strong correlation (r = 0.97) between JTA and mobility torsion of the vertebrae (Jones et al. 2020).

Joint verticality (JV) = absolute value of 180º – angle between postzygapophyses. JV estimates the relative importance of sagittal bending versus lateral bending of vertebral joints. Biomechanical testing using cadaveric bending experiments found a strong correlation (r = 0.77) between JV and the ratio of sagittal to lateral mobility of the vertebrae (Jones et al. 2020).

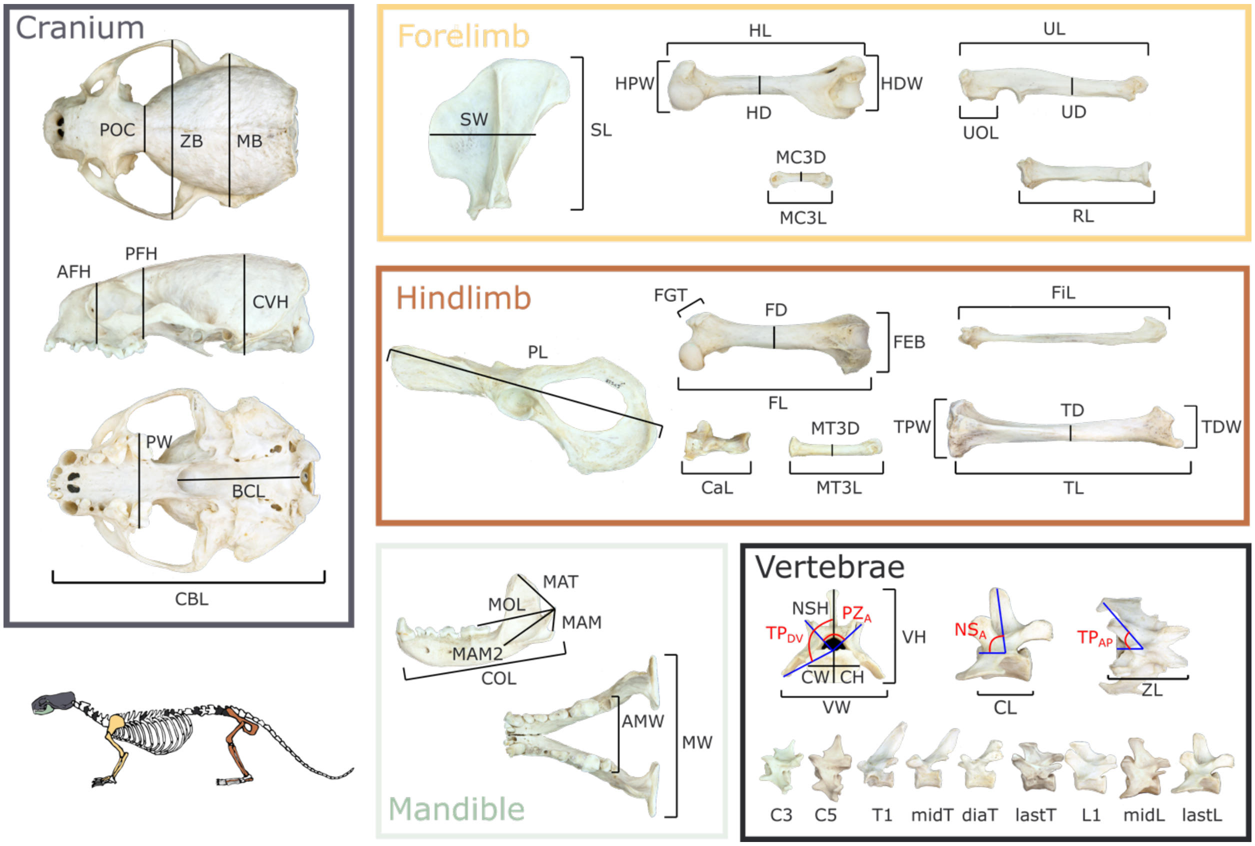

Fig. S1. Skeletal trait measurements used in this study. *Cranium*: CBL = condylobasal length, POC = postorbital constriction breadth, ZB = zygomatic breadth, MB = mastoid breadth, AFH = anterior facial height, PFH = posterior facial height, CVH = cranial vault height, PW = palate width, BCL = basicranial length. *Mandible*: MAT = moment arm of temporalis, MAM = moment arm of masseter, MAM2 = moment arm of masseter; MOL = molar out-lever; COL = canine out-lever; MW = mandibular width; AMW = anterior mandibular width. *Forelimb*: SL = scapula length; SW = scapula width; HL = humerus length; HD = humerus mid-shaft width; HPW = humerus proximal width; HDW = humerus distal width; UL = ulna length; UD = ulna mid-shaft width; UOL = ulnar olecranon length; RL = radius length; RD = radius mid-shaft width; MC3L = third metacarpel length; MC3W = third metacarpel width. *Hindlimb*: PL = pelvis length; FL = femur length; FD = femur mid-shaft width; FEB = femur distal width; FGT = height of the greater trochanter of the femur; TL = tibia length; TD = tibia mid-shaft width; TPW = tibia proximal width; TDW = tibia distal width; FiL = fibula length; CaL = calcaneus length; MT3L = third metatarsal length; MT3D = third metatarsal width. *Vertebrae*: VW = vertebrae width; VH = vertebrae height; CL = centrum length; CW = centrum width (posterior); CH = centrum height (posterior); ZL = inter-zygapophyseal length; NSH = neural spine height; TP_{DV} = transverse process dorsoventral projection; TP_{AP} = transverse process anteroposterior projection; PZ_A = angle between postzygapophyses; NSA_A = neural spine angle.

Table S1. PC loadings of skeletal traits. PC 1 and 2 explains 30.3% and 16.5% of the variance, respectively. Trait definitions are found in Figure S1.

trait	PC1	PC1	trait	PC1	PC1
lnCBL	0.050	-0.058	lnTPW	-0.040	0.066
lnPOC	0.039	0.005	lnTDW	-0.053	0.050
lnZB	-0.001	-0.025	lnFiL	0.130	0.118
lnMB	-0.062	-0.183	lnCaL	0.033	0.100
lnCVH	0.028	-0.051	lnMT3L	0.220	0.159
lnPW	0.074	-0.048	lnMT3D	0.038	0.123
lnBCL	0.081	-0.078	lnC3_VW	-0.045	-0.087
lnMAT	0.000	-0.098	lnC3_VH	0.042	-0.026
lnMAM	0.046	0.006	lnC3_CL	0.137	0.054
lnMAM2	0.028	0.009	lnC3_CW	-0.055	-0.012
lnMOL	0.017	0.021	lnC3_CH	0.019	-0.008
lnCOL	0.070	0.025	lnC3_ZL	0.118	0.021
lnMW	0.039	-0.088	lnC3_NSH	0.057	-0.039
lnAMW	0.033	-0.056	lnC3_TPDV	0.045	0.107
lnSL	0.007	0.143	lnC3_PZA	-0.017	-0.067
lnSW	-0.056	0.111	lnC3_NSA	-0.002	-0.048
lnHL	0.049	0.110	lnC3_TPAP	0.040	0.020
lnHD	-0.063	0.116	lnC5_VW	-0.081	-0.083
lnHPW	-0.052	0.042	lnC5_VH	0.041	0.027
lnHDW	-0.120	-0.015	lnC5_CL	0.090	-0.017
lnUL	0.053	0.206	lnC5_CW	-0.077	-0.054
lnUD	-0.129	-0.020	lnC5_CH	0.034	0.054
lnUOL	-0.056	0.054	lnC5_ZL	0.078	-0.024
lnRL	0.075	0.256	lnC5_NSH	0.045	0.011
lnRD	-0.060	0.248	lnC5_TPDV	0.067	0.075
lnMC3L	0.157	0.218	lnC5_PZA	-0.033	-0.067
lnMC3W	-0.019	0.099	lnC5_NSA	-0.044	-0.095
lnPL	-0.044	0.024	lnC5_TPAP	0.021	-0.003
lnFL	0.059	0.143	lnT1_VW	-0.067	-0.062
lnFD	-0.043	0.044	lnT1_VH	0.020	0.099
lnFEB	-0.047	0.035	lnT1_CL	0.062	-0.062
lnFGT	-0.135	0.047	lnT1_CW	-0.072	0.022
lnTL	0.124	0.114	lnT1_CH	-0.065	0.042
lnTD	0.023	0.159	lnT1_ZL	0.051	-0.047

trait	PC1	PC1	trait	PC1	PC1
lnT1_NSH	0.040	0.111	lnlastT_NSA	-0.147	0.094
lnT1_TPDV	0.165	0.051	lnL1_VW	-0.085	0.077
lnT1_PZA	-0.057	-0.035	lnL1_VH	-0.106	0.088
lnT1_NSA	-0.016	-0.010	lnL1_CL	0.093	-0.079
lnT1_TPAP	-0.040	0.027	lnL1_CW	-0.085	-0.003
lnmidT_VW	-0.052	-0.050	lnL1_CH	-0.110	0.031
lnmidT_VH	-0.040	0.129	lnL1_ZL	0.055	-0.072
lnmidT_CL	0.044	-0.120	lnL1_NSH	-0.104	0.117
lnmidT_CW	-0.053	0.010	lnL1_TPDV	0.124	0.130
lnmidT_CH	-0.082	0.086	lnL1_PZA	-0.059	-0.126
lnmidT_ZL	0.013	-0.114	lnL1_NSA	-0.160	0.095
lnmidT_NSH	-0.027	0.142	lnL1_TPAP	-0.119	0.054
lnmidT_TPDV	0.176	0.042	lnmidL_VW	-0.069	0.104
lnmidT_PZA	-0.118	-0.024	lnmidL_VH	-0.065	0.089
lnmidT_NSA	0.020	0.010	lnmidL_CL	0.128	-0.065
lnmidT_TPAP	-0.048	0.033	lnmidL_CW	-0.081	0.003
lntranT_VW	-0.052	-0.044	lnmidL_CH	-0.118	0.061
lntranT_VH	-0.113	0.074	lnmidL_ZL	0.090	-0.086
lntranT_CL	0.058	-0.091	lnmidL_NSH	-0.042	0.102
lntranT_CW	-0.060	0.000	lnmidL_TPDV	0.140	0.107
lntranT_CH	-0.103	0.064	lnmidL_PZA	-0.071	-0.137
lntranT_ZL	-0.012	-0.096	lnmidL_NSA	-0.156	0.028
lntranT_NSH	-0.117	0.076	lnmidL_TPAP	-0.260	0.023
lntranT_TPDV	0.142	0.086	lnlastL_VW	-0.049	0.068
lntranT_PZA	-0.089	-0.119	lnlastL_VH	-0.080	0.035
lntranT_NSA	0.014	0.032	lnlastL_CL	0.079	-0.078
lnlastT_VW	-0.071	-0.042	lnlastL_CW	-0.058	0.019
lnlastT_VH	-0.105	0.091	lnlastL_CH	-0.087	0.039
lnlastT_CL	0.079	-0.091	lnlastL_ZL	0.059	-0.100
lnlastT_CW	-0.081	-0.001	lnlastL_NSH	-0.070	0.040
lnlastT_CH	-0.114	0.039	lnlastL_TPDV	0.155	-0.054
lnlastT_ZL	0.046	-0.076	lnlastL_PZA	0.009	0.057
lnlastT_NSH	-0.101	0.118	lnlastL_NSA	-0.130	0.011
lnlastT_PZA	-0.055	-0.150	lnlastL_TPAP	-0.207	-0.013

Table S2. Pairwise tests among ecological regimes. Bolded P-values indicate significance.

Locomotor modes						
p-values	arboreal	cursorial	scansorial	semi-aquatic	semi-fossorial	terrestrial hunter
cursorial	**0.003**					
scansorial	0.517	**0.012**				
semi_aquatic	0.092	**0.001**	**0.014**			
semi_fossorial	**0.044**	**0.001**	**0.007**	0.867		
terrestrial hunter	0.079	**0.012**	**0.025**	**0.001**	**0.001**	
terrestrial non-hunter	0.358	0.183	0.673	0.130	0.057	0.090

Dietary ecologies						
p-values	large prey carnivore	med prey carnivore	small prey carnivore	herbivore	insectivore	omnivore
med prey carnivore	0.285					
small prey carnivore	0.175	0.207				
herbivore	0.150	0.332	**0.017**			
insectivore	0.115	0.127	**0.006**	0.752		
omnivore	0.739	0.195	**0.007**	0.249	0.202	
piscivore	**0.034**	0.102	**0.006**	0.527	0.386	**0.035**

Table S3. Relative weights of each theoretical functional proxy optimized in the combined adaptive landscape across all carnivorans and optimized among the seven locomotor adaptive landscapes. The values represent the mean weights of the traits calculated from the top 5% of landscapes. Functional proxy definitions are found in Table 1.

functional proxies	combined	arboreal	cursorial	scansorial	semi-aquatic	semi-fossorial	terrestrial hunter	terrestrial non-hunter
temMA	0.00	0.00	0.00	0.00	0.00	0.00	0.00	0.00
BI	0.00	0.00	0.04	0.00	0.00	0.00	0.00	0.00
HRI	0.00	0.00	0.00	0.00	0.00	0.00	0.00	0.00
HEI	0.00	0.00	0.00	0.00	0.28	0.40	0.00	0.00
OLI	0.00	0.00	0.00	0.00	0.01	0.03	0.00	0.00
MAN	0.03	0.00	0.56	0.00	0.00	0.00	0.07	0.21
CI	0.03	0.00	0.00	0.00	0.03	0.00	0.00	0.01
FRI	0.00	0.00	0.00	0.00	0.00	0.00	0.00	0.00
FEI	0.00	0.00	0.00	0.00	0.07	0.00	0.00	0.00
PES	0.00	0.00	0.00	0.00	0.01	0.00	0.00	0.01
URI	0.01	0.05	0.00	0.02	0.16	0.04	0.00	0.01
$SMA_{cervical}$	0.00	0.00	0.00	0.00	0.00	0.00	0.00	0.00
$JTA_{cervical}$	0.03	0.03	0.00	0.06	0.39	0.11	0.00	0.03
$JV_{cervical}$	0.03	0.03	0.02	0.00	0.00	0.00	0.07	0.03
$SMA_{thoracic}$	0.00	0.00	0.00	0.00	0.00	0.00	0.00	0.00
$JTA_{thoracic}$	0.00	0.00	0.00	0.00	0.00	0.00	0.00	0.01
$JV_{thoracic}$	0.41	0.18	0.06	0.29	0.01	0.04	0.45	0.04
SMA_{dia}	0.00	0.00	0.00	0.00	0.00	0.00	0.00	0.00
JTA_{dia}	0.03	0.03	0.00	0.04	0.04	0.37	0.00	0.60
JV_{dia}	0.03	0.03	0.04	0.02	0.00	0.00	0.02	0.00
SMA_{lumbar}	0.00	0.00	0.00	0.00	0.00	0.00	0.00	0.00
JTA_{lumbar}	0.00	0.00	0.00	0.00	0.00	0.00	0.00	0.03
JV_{lumbar}	0.38	0.68	0.29	0.56	0.00	0.00	0.39	0.03
Z-score	0.62	0.64	0.83	0.63	0.73	0.71	0.70	0.61

Table S4. Pairwise significance tests among dietary adaptive landscapes. Top triangle: number of landscape models shared in the top 1% between the paired groups. Bottom triangle: P-values for difference between groups. Bolded P-values indicate significance.

	large prey carnivore	med prey carnivore	small prey carnivore	herbivore	insectivore	omnivore	piscivore
large prey carnivore	-	4	6	5	5	8	0
medium prey carnivore	0.222	-	6	1	8	3	0
small prey carnivore	0.333	0.462	-	3	5	5	0
herbivore	0.278	0.077	0.333	-	40	7	1
insectivore	0.278	0.615	0.556	0.656	-	7	21
omnivore	0.444	0.231	0.556	0.115	**0.045**	-	0
piscivore	**0.001**	**0.001**	**0.001**	**0.016**	0.135	**0.001**	-

Table S5. Relative weights of each functional proxy optimized among the dietary adaptive landscapes. The values represent the mean weights of the traits calculated from the top 5% of landscapes. Functional proxy definitions are found in Table 1.

functional traits	large prey carnivore	med prey carnivore	small prey carnivore	herbivore	insectivore	omnivore	piscivore
temMA	0.00	0.02	0.00	0.03	0.00	0.00	0.00
BI	0.00	0.00	0.00	0.00	0.00	0.00	0.00
HRI	0.00	0.00	0.00	0.00	0.04	0.00	0.00
HEI	0.00	0.02	0.00	0.03	0.11	0.00	0.32
OLI	0.00	0.00	0.00	0.00	0.00	0.00	0.06
MAN	0.29	0.02	0.03	0.00	0.00	0.02	0.00
CI	0.00	0.00	0.00	0.00	0.00	0.02	0.03
FRI	0.00	0.00	0.00	0.00	0.00	0.00	0.00
FEI	0.00	0.00	0.00	0.00	0.00	0.00	0.11
PES	0.00	0.00	0.00	0.00	0.00	0.00	0.00
URI	0.00	0.02	0.00	0.21	0.21	0.00	0.22
$SMA_{cervical}$	0.00	0.00	0.00	0.00	0.00	0.00	0.00
$JTA_{cervical}$	0.00	0.02	0.00	0.03	0.16	0.02	0.22
$JV_{cervical}$	0.01	0.04	0.03	0.03	0.00	0.02	0.00
$SMA_{thoracic}$	0.00	0.00	0.00	0.00	0.00	0.00	0.00
$JTA_{thoracic}$	0.00	0.00	0.00	0.02	0.01	0.00	0.00
$JV_{thoracic}$	0.28	0.71	0.58	0.07	0.14	0.23	0.00
SMA_{dia}	0.00	0.00	0.00	0.00	0.00	0.00	0.00
JTA_{dia}	0.00	0.04	0.00	0.20	0.19	0.02	0.03
JV_{dia}	0.03	0.02	0.06	0.00	0.00	0.02	0.00
SMA_{lumbar}	0.00	0.00	0.00	0.00	0.00	0.00	0.00
JTA_{lumbar}	0.00	0.00	0.00	0.00	0.01	0.00	0.00
JV_{lumbar}	0.39	0.10	0.31	0.38	0.13	0.64	0.00
Z-score	0.71	0.61	0.69	0.57	0.58	0.64	0.85

Table S6. Relative weights of each functional proxy optimized among the carnivoran familial adaptive landscapes. The values represent the mean weights of the traits calculated from the top 5% of landscapes. Functional proxy definitions are found in Table 1.

functional traits	combined	Feli	Vive	Hyae	Eupl	Herp	Cani	Ursi	Meph	Proc	Must
temMA	0.00	0.00	0.00	0.00	0.00	0.00	0.00	0.00	0.03	0.00	0.04
BI	0.00	0.00	0.00	0.50	0.00	0.00	0.00	0.00	0.00	0.00	0.00
HRI	0.00	0.00	0.00	0.00	0.00	0.00	0.00	0.08	0.00	0.00	0.00
HEI	0.00	0.00	0.00	0.00	0.00	0.00	0.00	0.38	0.38	0.00	0.13
OLI	0.00	0.00	0.00	0.00	0.00	0.00	0.00	0.00	0.00	0.00	0.00
MAN	0.03	0.05	0.00	0.50	0.00	0.04	0.33	0.00	0.00	0.00	0.00
CI	0.03	0.00	0.00	0.00	0.04	0.04	0.00	0.00	0.03	0.00	0.04
FRI	0.00	0.00	0.00	0.00	0.00	0.00	0.00	0.00	0.00	0.00	0.00
FEI	0.00	0.00	0.00	0.00	0.00	0.00	0.00	0.00	0.00	0.00	0.02
PES	0.00	0.00	0.00	0.00	0.00	0.04	0.00	0.00	0.00	0.00	0.01
URI	0.01	0.00	0.00	0.00	0.00	0.00	0.00	0.14	0.03	0.00	0.17
$SMA_{cervical}$	0.00	0.00	0.00	0.00	0.00	0.00	0.00	0.00	0.00	0.00	0.00
$JTA_{cervical}$	0.03	0.00	0.00	0.00	0.13	0.04	0.00	0.00	0.08	0.00	0.18
$JV_{cervical}$	0.03	0.02	0.07	0.00	0.00	0.00	0.05	0.00	0.00	0.00	0.00
$SMA_{thoracic}$	0.00	0.00	0.00	0.00	0.00	0.00	0.00	0.00	0.00	0.00	0.00
$JTA_{thoracic}$	0.00	0.00	0.00	0.00	0.00	0.00	0.00	0.17	0.00	0.00	0.00
$JV_{thoracic}$	0.41	0.55	0.36	0.00	0.75	0.79	0.13	0.00	0.03	0.13	0.22
SMA_{dia}	0.00	0.00	0.00	0.00	0.00	0.00	0.00	0.00	0.00	0.00	0.00
JTA_{dia}	0.03	0.00	0.00	0.00	0.04	0.04	0.00	0.24	0.38	0.00	0.13
JV_{dia}	0.03	0.07	0.07	0.00	0.00	0.00	0.02	0.00	0.00	0.00	0.00
SMA_{lumbar}	0.00	0.00	0.00	0.00	0.00	0.00	0.00	0.00	0.00	0.00	0.00
JTA_{lumbar}	0.00	0.00	0.00	0.00	0.00	0.04	0.00	0.00	0.00	0.00	0.00
JV_{lumbar}	0.38	0.32	0.50	0.00	0.04	0.00	0.47	0.00	0.03	0.88	0.05
Z-score	0.62	0.69	0.67	0.90	0.67	0.67	0.81	0.79	0.67	0.77	0.62

Table S7. Pairwise significance tests among familial adaptive landscapes. Top triangle: number of landscape models shared in the top 1% between the paired groups. Bottom triangle: P-values for difference between groups. Bolded P-values indicate significance.

	Feli	Vive	Hyae	Eupl	Herp	Cani	Ursi	Meph	Proc	Must
Feli	-	7	0	5	6	35	0	0	8	1
Vive	0.200	-	0	8	10	7	0	0	7	1
Hyae	**0.001**	**0. 001**	-	0	0	0	0	0	0	0
Eupl	0.143	0.727	**0. 001**	-	8	5	0	0	7	1
Herp	0.171	0.909	**0. 001**	1.000	-	6	0	0	7	1
Cani	1.000	0.636	**0. 001**	0.625	0.600	-	0	0	8	1
Ursi	**0. 001**	**0. 001**	0.059	**0. 001**	**0. 001**	**0. 001**	-	0	0	0
Meph	**0. 001**	**0. 001**	**0. 001**	**0. 001**	**0. 001**	**0. 001**	**0. 001**	-	0	58
Proc	0.229	0.636	**0. 001**	0.875	0.700	0.084	**0. 001**	**0. 001**	-	4
Must	**0.029**	0.091	**0. 001**	0.125	0.100	**0.011**	**0. 001**	0.630	0.267	-

Supplementary Results

Performance surfaces based on theoretical morphology

The performance surfaces generated based on theoretical morphologies showed similar trends as the performance surfaces generated using the empirical data. In the skull, mechanical advantage of jaw closure tends to be greatest in the bottom of morphospace, with temporalis mechanical advantage (temMA) highest towards the bottom-left (-PC1, -PC2) and declining towards the top-right (+PC1, +PC2) whereas masseter mechanical advantage (masMA) is highest towards the bottom-right (+PC1, -PC2) and declines towards the top-left (-PC1, +PC2) (Fig. S2).

In the forelimb, there is a trade-off between limb elongation and elbow robustness: functional proxies of scapula (SI), radius (BI), and metacarpal (MAN) elongation are highest in the top-right (+PC1, +PC2) of morphospace and transitions towards increased robustness diagonally to the bottom-left (-PC1, -PC2). Proxies associated with increased mechanical advantage of elbow extension (OLI) and attachment sites for forearm flexor, pronator, and supinator muscles on the humeral epicondyles (HEI) and ulna (URI) exhibit the opposite pattern (i.e., highest in the bottom-left and lowest in the top-right). Overall robustness of humerus (HRI) is highest on the left side of the morphospace (-PC1) and transitions towards increased elongation to the right (+PC1), following a similar distribution as the latter indices. The hindlimb also exhibits a trade-off between elongation and robustness: indices of tibial (CI) and metatarsal (PES) elongation tend to be highest in the right side (+PC1, with CI also trending towards -PC2) and transitions to increased robustness towards to the left side (-PC2), whereas indices for femoral (FEI, FRI) and tibial (TRI) robustness tend to be highest on the left side (-PC1) but transitions towards less robustness on PC2 (FEI and FRI is highest towards -PC2 and TRI is highest towards +PC2).

In the vertebral joints, the performance surfaces show a trade-off between joint torsional angle (JTA) as a proxy for range of rotational motion and joint verticality (JV) as a proxy for sagittal mobility. For the cervical, diaphragmatic, and lumbar joints, JTA is highest in the bottom-left of the morphospace (-PC1, -PC2) and declines diagonally to the top-right (+PC1, +PC2), whereas JV exhibits the opposite pattern (i.e., highest in the top-right and lowest in the bottom-left). The thoracic vertebra exhibits similar JTA and JV distributions but in the horizontal plane (i.e., highest JTA in the left side of morphospace and highest JV in the right). In all vertebral joints, second moment of area (SMA) as a proxy for stiffness tends to be greatest towards the top-left of morphospace (-PC1, +PC2) and declines towards the right side of morphospace (+PC1).

Optimized adaptive landscapes based on theoretical morphology

After summation of all performance surfaces based on optimized weights, we found that the combined adaptive landscape is heavily weighted for sagittal mobility of the pre-diaphragmatic thoracic region ($w_{JV} = 0.71$; Fig. S3A; Table S8). When adaptive landscapes are optimized by locomotor ecologies, we found different degrees to which the 27 functional proxies are incorporated among the different adaptive landscapes. The cursorial landscape is characterized by evenly weighted functional proxies associated with increased sagittal mobility of the vertebral column ($w_{JV} = 0.13$–0.20 for the cervical, pre-diaphragmatic thoracic, diaphragmatic, and lumbar regions) and lengthening of the forelimb, particularly in the scapula ($w_{SI} = 0.09$), radius ($w_{BI} = 0.13$), and metacarpal ($w_{MAN} = 0.07$) (Fig. S3C; Table S8). This landscape is significantly different from all other locomotor landscapes except with the terrestrial non-hunter landscape (Table S9). Semi-aquatic and semi-fossorial landscapes do not significantly differ from one another ($P = 0.917$; Table S9), and both are weighted for increased

joint torsion in the cervical (w_{JTA} = 0.25), diaphragmatic (w_{JTA} = 0.09), and lumbar (w_{JTA} = 0.22) regions; larger femoral epicondyles (w_{FEI} = 0.25); and enlarged olecranon processes (w_{OLI} = 0.09) (Fig. S3E, F; Table S9). The remaining locomotor landscapes do not significantly differ from one another (Table S9) and are heavily weighted for sagittal mobility of the pre-diaphragmatic thoracic region (w_{JV} = 0.51–0.78; Fig. S3; Table S8).

When adaptive landscapes are optimized by dietary ecologies, we found that differences in landscapes appear associated with carnivory (Fig. S4; Table S10). Adaptive landscapes based on diets that largely consists of terrestrial vertebrate prey (i.e., carnivory and omnivory) are heavily weighted for sagittal mobility of the pre-diaphragmatic thoracic region (w_{JV} = 0.66–0.70; Fig. S4; Table S11). In contrast, adaptive landscapes based on diets that do not incorporate terrestrial vertebrate prey (i.e., herbivory, insectivory, and piscivory) are characterized by evenly weighted functional proxies associated with increased joint torsion in the cervical and lumbar regions and larger femoral epicondyles (Fig. S4; Table S11).

Lastly, we found different adaptive landscapes when they are optimized by family. Adaptive landscapes for felids, viverrids, euplerids, herpestids, and procyonids are not significantly different from each other and all remain heavily weighted for sagittal mobility of the pre-diaphragmatic thoracic region (w_{JV} = 0.52–0.79; Fig. S5; Table S12; Table S13). The canid landscape resembles the cursorial landscape with similar weights for increased sagittal mobility of the pre-diaphragmatic thoracic (w_{JV} = 0.36) and diaphragmatic (w_{JV} = 0.23) joints and lengthening of the scapula (w_{SI} = 0.14) (Fig. S5G; Table S12); however, it does not significantly differ from the above family-specific landscapes (Table S13). The mephitid and mustelid landscapes resemble semi-aquatic and semi-fossorial landscapes with similar weights for larger femoral epicondyles (w_{FEI} = 0.21–0.34) and increased joint torsion in the cervical (w_{JTA}

$= 0.20–0.29$) and lumbar ($w_{JTA} = 0.16–0.19$) joints (Fig. S5I, K), but only the mephitid landscape significantly differs from all other families (Table S13). The hyaenid landscape weighted heavily for increased stiffness of all the cervical joints ($w_{SMA} = 0.40$) and elongation of the radius ($w_{BI} = 0.22$) (Fig. S5D), whereas the ursid landscape weighted heavily among increased stiffness of all the vertebral joints ($\sum w_{SMA} = 0.53$) and increased robustness of the humerus ($w_{HRI} = 0.18$) and tibia ($w_{TRI} = 0.20$) (Fig. S5H). Both hyaenid and ursid landscapes significantly differ with all other family-specific landscapes (Table S13).

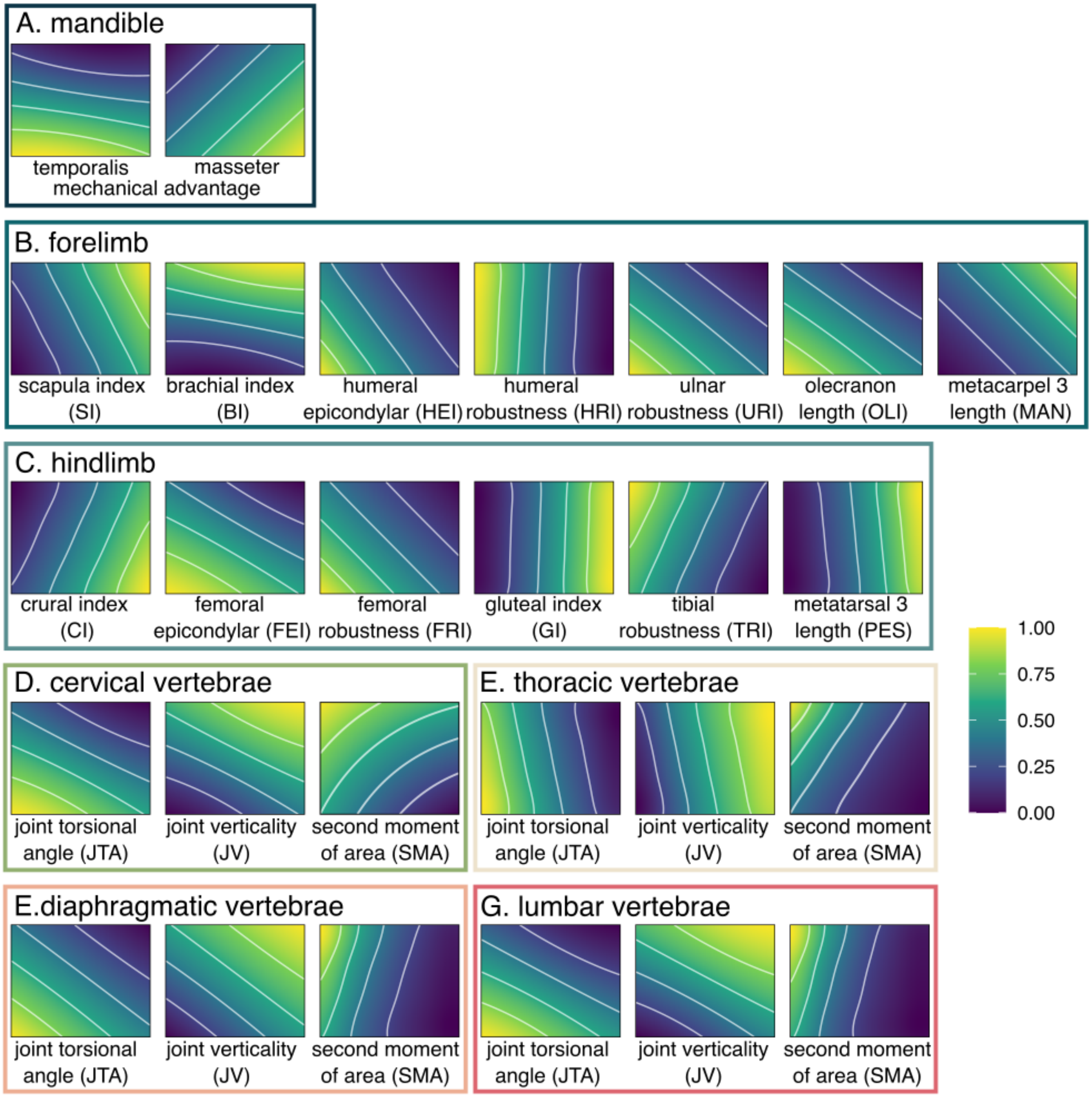

Fig. S2. Performance surfaces for each functional proxy based on theoretical morphology. Color represents height on the performance surface. See Table 1 for functional proxies definitions.

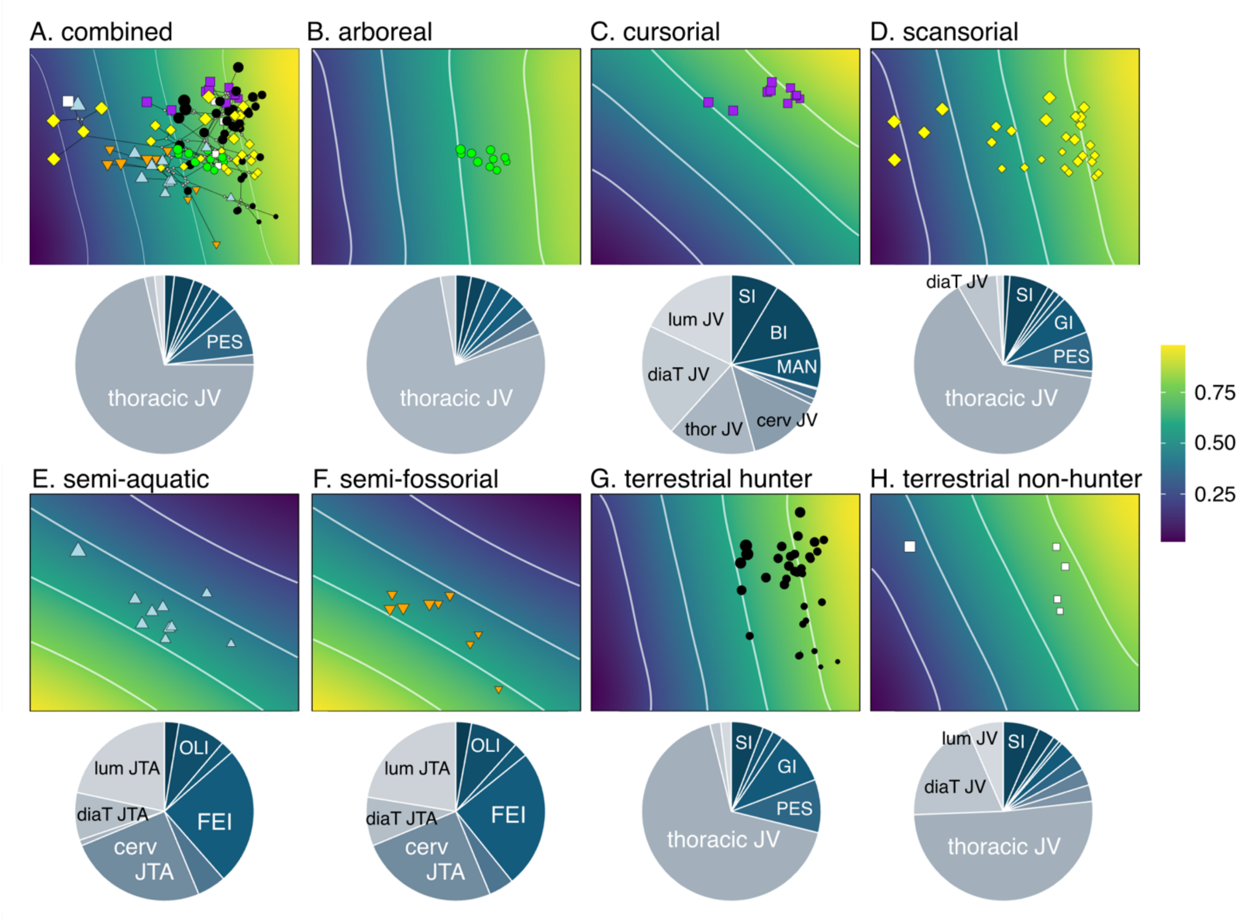

Fig. S3. Adaptive landscapes optimized for all carnivorans and each locomotor group based on theoretical morphology. Landscapes were produced by combining the performance surfaces and optimizing their weightings to maximize the height of the landscape at the group mean. Pie charts show the relative weights of each performance surface on each landscape (Table S8 shows breakdown of weights). Functional proxies with weights > 0.07 were labeled. Table S9 shows statistical tests comparing adaptive landscapes among locomotor groups. Size of points are scaled to estimated body size based on the geometric mean of all measurements.

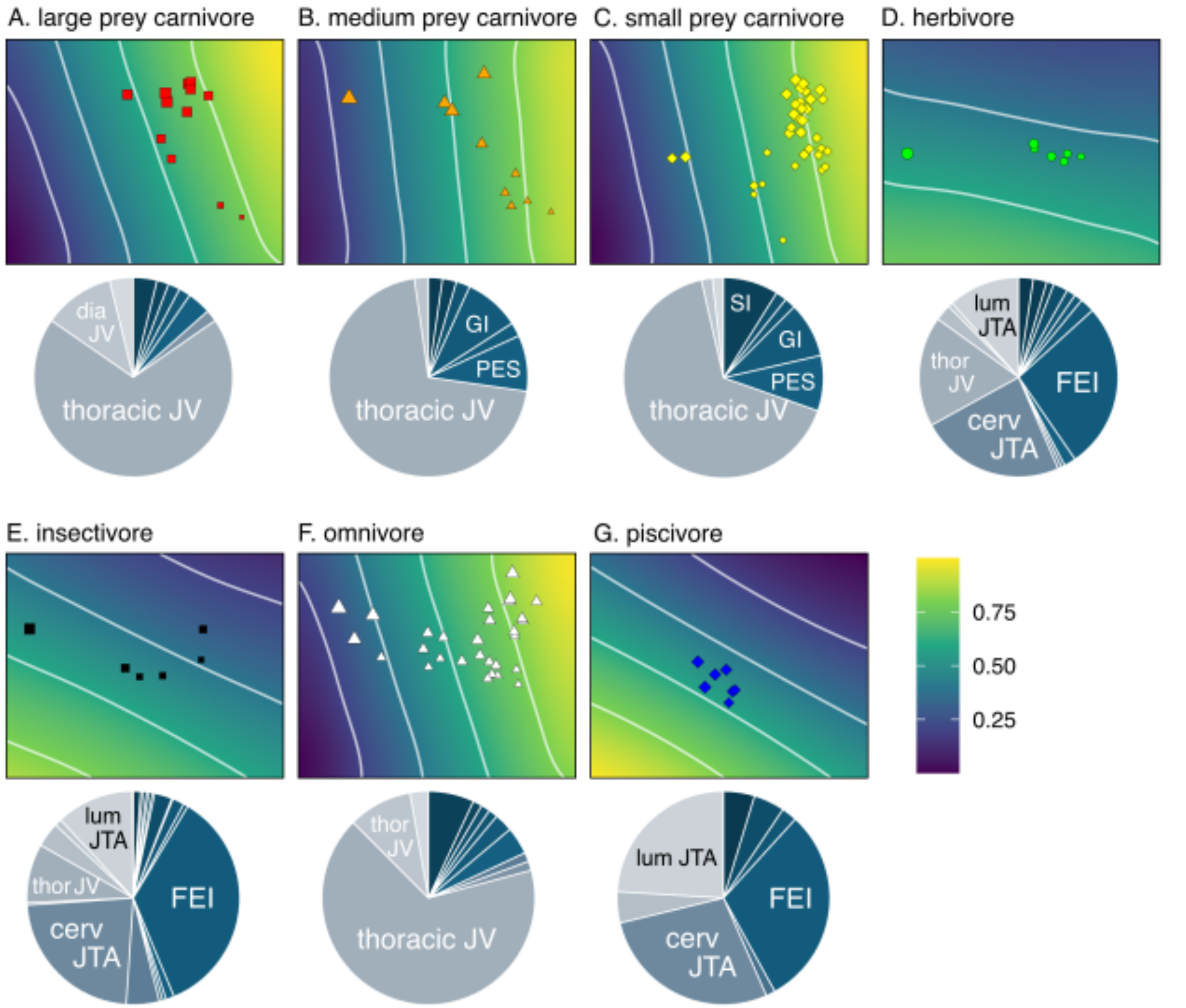

Fig. S4. Adaptive landscapes optimized for each dietary group based on theoretical morphology. Adaptive landscapes were produced by combining the performance surfaces and optimizing their weightings to maximize the height of the landscape at the group mean. Pie charts show the relative weighting of each performance surface on each adaptive landscape (see Table S11 for full breakdown of optima weights). Functional indices with weights > 0.09 were labeled. Statistical tests comparing adaptive landscapes among dietary groups can be found in Table S10.

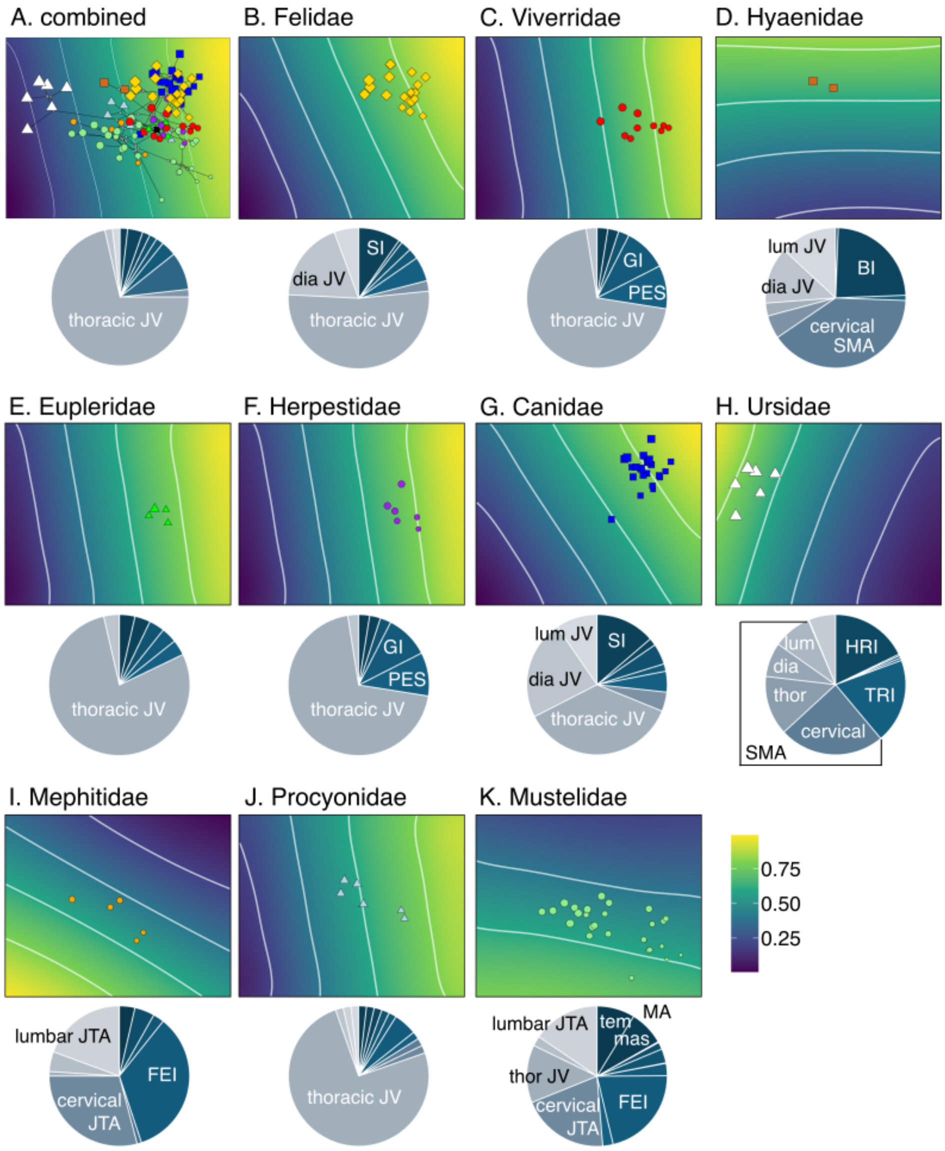

Fig. S5. Adaptive landscapes optimized for all carnivorans and each family based on theoretical morphology. Landscapes were produced by combining the performance surfaces and optimizing their weightings to maximize the height of the landscape at the group mean. Pie charts show the relative weights of each performance surface on each adaptive landscape (Table S12 shows breakdown of weights). Functional proxies with weights > 0.09 were labeled. Table S13 shows statistical tests comparing adaptive landscapes among families. Size of points are scaled to estimated body size based on the geometric mean of all measurements.

Table S8. Relative weights of each theoretical functional proxy optimized in the combined adaptive landscape across all carnivorans and optimized among the seven locomotor adaptive landscapes based on theoretical morphology. The values represent the mean weights of the traits calculated from the top 5% of landscapes. Functional proxy definitions are found in Table 1.

functional proxies	combined	arboreal	cursorial	scansorial	semi-aquatic	semi-fossorial	terrestrial hunter	terrestrial non-hunter
temMA	0.00	0.00	0.00	0.00	0.03	0.03	0.00	0.00
masMA	0.02	0.03	0.00	0.01	0.00	0.00	0.00	0.00
SI	0.04	0.03	0.10	0.07	0.00	0.00	0.09	0.06
BI	0.02	0.00	0.15	0.01	0.00	0.00	0.00	0.07
HRI	0.00	0.00	0.00	0.00	0.00	0.00	0.00	0.00
HEI	0.00	0.00	0.00	0.00	0.00	0.00	0.00	0.00
OLI	0.00	0.00	0.00	0.00	0.09	0.09	0.00	0.00
MAN	0.02	0.00	0.10	0.01	0.00	0.00	0.02	0.02
CI	0.02	0.03	0.00	0.01	0.00	0.00	0.02	0.01
FRI	0.00	0.00	0.00	0.00	0.02	0.03	0.00	0.00
GI	0.04	0.03	0.03	0.07	0.00	0.00	0.09	0.03
FEI	0.00	0.03	0.00	0.00	0.25	0.25	0.00	0.00
TRI	0.00	0.00	0.00	0.00	0.00	0.00	0.00	0.00
PES	0.09	0.03	0.05	0.08	0.00	0.00	0.09	0.05
URI	0.00	0.00	0.00	0.00	0.05	0.05	0.00	0.00
$SMA_{cervical}$	0.00	0.00	0.00	0.00	0.00	0.00	0.00	0.00
$JTA_{cervical}$	0.00	0.03	0.00	0.00	0.25	0.25	0.00	0.00
$JV_{cervical}$	0.02	0.00	0.14	0.01	0.00	0.00	0.00	0.05
$SMA_{thoracic}$	0.00	0.00	0.00	0.00	0.00	0.00	0.00	0.00
$JTA_{thoracic}$	0.00	0.00	0.00	0.00	0.00	0.00	0.00	0.00
$JV_{thoracic}$	0.71	0.78	0.14	0.67	0.01	0.00	0.66	0.56
SMA_{dia}	0.00	0.00	0.00	0.00	0.00	0.00	0.00	0.00
JTA_{dia}	0.00	0.00	0.00	0.00	0.09	0.09	0.00	0.00
JV_{dia}	0.02	0.03	0.15	0.03	0.00	0.00	0.02	0.10
SMA_{lumbar}	0.00	0.00	0.00	0.00	0.00	0.00	0.00	0.00
JTA_{lumbar}	0.00	0.03	0.00	0.00	0.22	0.22	0.00	0.00
JV_{lumbar}	0.02	0.00	0.15	0.01	0.00	0.00	0.02	0.06
Z-score	0.69	0.69	0.80	0.68	0.70	0.76	0.81	0.63

Table S9. Pairwise significance tests among locomotor adaptive landscapes based on theoretical morphology. Top triangle: number of landscape models shared in the top 5% between the paired groups. Bottom triangle: P-values for difference between groups. Bolded P-values indicate significance.

	arboreal	cursorial	scansorial	semi-aquatic	semi-fossorial	terrestrial hunter	terrestrial non-hunter
arboreal	-	4	7	0	0	6	6
cursorial	0.400	-	10	0	0	7	34
scansorial	0.700	**0.033**	-	0	0	14	15
semi-aquatic	**0.001**	**0.001**	**0.001**	-	88	0	0
semi-fossorial	**0.001**	**0.001**	**0.001**	0.917	-	0	0
terrestrial hunter	0.600	**0.023**	0.778	**0.001**	**0.001**	-	12
terrestrial nonhunter	0.600	0.114	0.833	**0.001**	**0.001**	0.857	-

Table S10. Pairwise significance tests among dietary adaptive landscapes based on theoretical morphology. Top triangle: number of landscape models shared in the top 1% between the paired groups. Bottom triangle: P-values for difference between groups. Bolded P-values indicate significance.

	large prey carnivore	med prey carnivore	small prey carnivore	herbivore	insectivore	omnivore	piscivore
large prey carnivore	-	5	7	3	0	11	0
med prey carnivore	0.455	-	9	7	2	7	0
small prey carnivore	0.636	0.643	-	4	0	11	0
herbivore	0.273	0.500	0.286	-	196	4	63
insectivore	**0.001**	0.143	**0.001**	0.616	-	0	62
omnivore	1.000	0.500	0.786	**0.013**	**0.001**	-	0
piscivore	**0.001**	**0.001**	**0.001**	0.198	0.227	**0.001**	-

Table S11. Relative weights of each functional proxy optimized among the dietary adaptive landscapes based on theoretical morphology. The values represent the mean weights of the traits calculated from the top 5% of landscapes. Functional proxy definitions are found in Table 1.

functional proxies	large prey carnivore	med prey carnivore	small prey carnivore	herbivore	insectivore	omnivore	piscivore
temMA	0.00	0.00	0.00	0.03	0.01	0.00	0.03
masMA	0.00	0.02	0.00	0.02	0.00	0.00	0.00
SI	0.05	0.02	0.09	0.01	0.00	0.08	0.00
BI	0.02	0.00	0.00	0.00	0.01	0.01	0.00
HRI	0.00	0.00	0.00	0.00	0.01	0.00	0.00
HEI	0.00	0.00	0.00	0.00	0.00	0.00	0.00
OLI	0.00	0.00	0.00	0.06	0.07	0.00	0.09
MAN	0.02	0.00	0.02	0.00	0.00	0.01	0.00
CI	0.00	0.05	0.02	0.02	0.00	0.01	0.00
FRI	0.00	0.00	0.00	0.01	0.02	0.00	0.03
GI	0.02	0.09	0.09	0.02	0.01	0.03	0.00
FEI	0.00	0.02	0.00	0.20	0.23	0.00	0.25
TRI	0.00	0.00	0.00	0.00	0.01	0.00	0.00
PES	0.02	0.09	0.09	0.02	0.00	0.07	0.00
URI	0.00	0.00	0.00	0.03	0.04	0.00	0.05
$SMA_{cervical}$	0.00	0.00	0.00	0.01	0.07	0.01	0.00
$JTA_{cervical}$	0.00	0.02	0.00	0.20	0.20	0.00	0.25
$JV_{cervical}$	0.02	0.00	0.00	0.00	0.00	0.01	0.00
$SMA_{thoracic}$	0.00	0.00	0.00	0.00	0.00	0.00	0.00
$JTA_{thoracic}$	0.00	0.00	0.00	0.00	0.00	0.00	0.00
$JV_{thoracic}$	0.73	0.68	0.66	0.15	0.08	0.67	0.00
SMA_{dia}	0.00	0.00	0.00	0.00	0.00	0.00	0.00
JTA_{dia}	0.00	0.00	0.00	0.06	0.07	0.00	0.09
JV_{dia}	0.09	0.02	0.02	0.00	0.00	0.07	0.00
SMA_{lumbar}	0.00	0.00	0.00	0.00	0.00	0.00	0.00
JTA_{lumbar}	0.00	0.00	0.00	0.16	0.15	0.00	0.22
JV_{lumbar}	0.02	0.00	0.02	0.00	0.00	0.01	0.00
Z-score	0.72	0.70	0.79	0.59	0.57	0.68	0.75

Table S12. Relative weights of each functional proxy optimized among the carnivoran familial adaptive landscapes based on theoretical morphology. The values represent the mean weights of the traits calculated from the top 5% of landscapes. Functional proxy definitions are found in Table 1.

functional proxies	Nand	Feli	Vive	Hyae	Eupl	Herp	Cani	Ursi	Meph	Ailu	Proc	Must
temMA	0.00	0.00	0.00	0.00	0.00	0.00	0.00	0.00	0.03	0.02	0.00	0.08
masMA	0.04	0.00	0.02	0.00	0.03	0.03	0.00	0.00	0.00	0.02	0.02	0.06
SI	0.04	0.09	0.05	0.00	0.03	0.03	0.14	0.00	0.00	0.02	0.02	0.00
BI	0.00	0.02	0.00	0.33	0.00	0.00	0.03	0.00	0.00	0.00	0.02	0.00
HRI	0.00	0.00	0.00	0.00	0.00	0.00	0.00	0.16	0.00	0.00	0.00	0.00
HEI	0.00	0.00	0.00	0.00	0.00	0.00	0.00	0.01	0.00	0.00	0.00	0.00
OLI	0.00	0.00	0.00	0.00	0.00	0.00	0.00	0.00	0.08	0.02	0.02	0.03
MAN	0.00	0.04	0.00	0.00	0.00	0.00	0.06	0.00	0.00	0.00	0.00	0.00
CI	0.04	0.00	0.02	0.00	0.03	0.03	0.00	0.00	0.00	0.02	0.02	0.03
FRI	0.00	0.00	0.00	0.00	0.00	0.00	0.00	0.01	0.02	0.00	0.00	0.00
GI	0.04	0.02	0.09	0.00	0.09	0.10	0.02	0.00	0.00	0.02	0.02	0.02
FEI	0.00	0.00	0.00	0.00	0.00	0.00	0.00	0.01	0.26	0.06	0.02	0.20
TRI	0.00	0.00	0.00	0.03	0.00	0.00	0.00	0.19	0.00	0.00	0.00	0.00
PES	0.04	0.06	0.11	0.00	0.03	0.10	0.05	0.00	0.00	0.02	0.02	0.02
URI	0.00	0.00	0.00	0.00	0.00	0.00	0.00	0.00	0.04	0.00	0.00	0.01
$SMA_{cervical}$	0.00	0.00	0.00	0.39	0.00	0.00	0.00	0.24	0.00	0.00	0.02	0.00
$JTA_{cervical}$	0.00	0.00	0.00	0.00	0.00	0.00	0.00	0.00	0.26	0.06	0.02	0.22
$JV_{cervical}$	0.00	0.05	0.00	0.07	0.00	0.00	0.07	0.00	0.00	0.00	0.02	0.00
$SMA_{thoracic}$	0.00	0.00	0.00	0.00	0.00	0.00	0.00	0.13	0.00	0.00	0.00	0.00
$JTA_{thoracic}$	0.00	0.00	0.00	0.00	0.00	0.00	0.00	0.08	0.00	0.00	0.00	0.00
$JV_{thoracic}$	0.79	0.55	0.68	0.03	0.75	0.70	0.36	0.00	0.01	0.72	0.77	0.12
SMA_{dia}	0.00	0.00	0.00	0.00	0.00	0.00	0.00	0.10	0.00	0.00	0.00	0.00
JTA_{dia}	0.00	0.00	0.00	0.00	0.00	0.00	0.00	0.01	0.08	0.02	0.02	0.03
JV_{dia}	0.04	0.11	0.02	0.05	0.03	0.03	0.17	0.00	0.00	0.02	0.02	0.00
SMA_{lumbar}	0.00	0.00	0.00	0.00	0.00	0.00	0.00	0.07	0.00	0.00	0.00	0.00
JTA_{lumbar}	0.00	0.00	0.00	0.00	0.00	0.00	0.00	0.00	0.21	0.02	0.02	0.18
JV_{lumbar}	0.00	0.06	0.00	0.10	0.00	0.00	0.10	0.00	0.00	0.00	0.02	0.00
Z-score	0.73	0.81	0.78	0.69	0.74	0.78	0.83	0.89	0.70	0.66	0.62	0.68

Table S13. Pairwise significance tests among familial adaptive landscapes based on theoretical morphology. Top triangle: number of landscape models shared in the top 1% between the paired groups. Bottom triangle: P-values for difference between groups. Bolded P-values indicate significance.

	Nand	Feli	Vive	Hyae	Eupl	Herp	Cani	Ursi	Meph	Ailu	Proc	Must
Nand	-	5	7	0	7	7	5	0	0	7	7	1
Feli	0.714	-	7	0	5	6	35	0	0	5	8	1
Vive	1.000	0.200	-	0	8	10	7	0	0	7	7	1
Hyae	**0.001**	**0.001**	**0.001**	-	0	0	0	0	0	0	0	0
Eupl	1.000	0.143	0.727	**0.001**	-	8	5	0	0	7	7	1
Herp	1.000	0.171	0.909	**0.001**	1.000	-	6	0	0	7	7	1
Cani	0.714	1.000	0.636	**0.001**	0.625	0.600	-	0	0	5	8	1
Ursi	**0.001**	**0.001**	**0.001**	0.059	**0.001**	**0.001**	**0.001**	-	0	0	0	0
Meph	**0.001**	**0.001**	**0.001**	**0.001**	**0.001**	**0.001**	**0.001**	**0.001**	-	0	0	58
Ailu	1.000	0.143	0.636	**0.001**	0.875	0.700	0.053	**0.001**	**0.001**	-	12	8
Proc	1.000	0.229	0.636	**0.001**	0.875	0.700	0.084	**0.001**	**0.001**	0.750	-	4
Must	0.143	**0.029**	0.091	**0.001**	0.125	0.100	**0.011**	**0.001**	0.630	0.500	0.267	-

Sensitivity Analyses

Our adaptive landscape results may potentially be affected by (1) removing the effects of size on the morphological traits in creating the morphospace and (2) the coarse increments of partition weights. Therefore, we conducted a series of sensitivity analyses to investigate these potential problems. We used theoretical data to simplify the interpretations of these Sensitivity Analyses.

Sensitivity Analyses 1: concerns with removing the effects of size in creating the morphospace

We conducted sensitivity analyses to investigate whether removing the effects of size on the morphological traits in creating the morphospace may potentially influence our performance surfaces and adaptive landscape results. Although it is standard practice to remove the effects of size on morphology analyses, size may be an important predictor of functional performance across Carnivora. Terrestrial carnivorans exhibit a diverse range in body sizes from the 100 g least weasel to 800 kg polar bear. Size effects may therefore facilitate distinct ecological adaptations. We performed a PCA with the same 136 linear and angular measurements but with no size correcting to create the morphospace of the 109 terrestrial carnivoran species. We used the first two PC axes (PC 1 and PC 2 captured 92.4% and 0.02% of the variance, respectively) to create the morphospace (Fig. S6). We then used the same procedure described in the Supplementary Methods to create performance surfaces and optimized adaptive landscapes. We found that functional trade-offs and covariation patterns in the performance surfaces remain similar to the performance surfaces created using size-corrected traits (Fig. S7). The adaptive landscapes optimized by locomotion, diet, and family also exhibit similar topologies and optimized weights as the adaptive landscapes created using size-corrected traits (Fig. S8; Table S14–11).

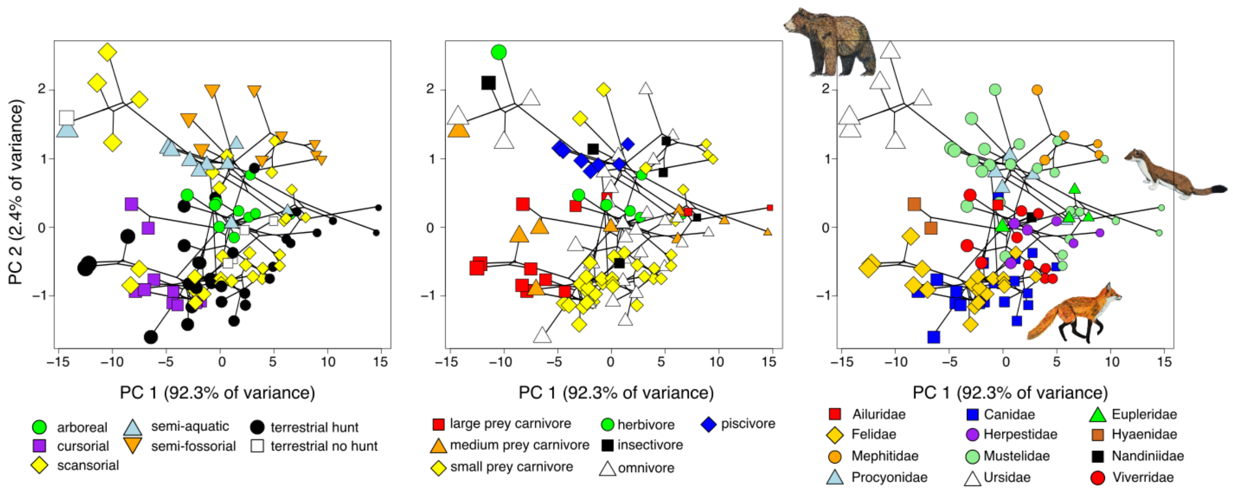

Fig. S6. Phylomorphospace of the carnivoran skeletal system defined by principal components (PCs) 1 and 2. PCA was conducted using 136 non-size corrected linear and angular measurements that capture morphological variation across the skull, appendicular, and axial skeletons. Size of points are scaled to estimated body size based on the geometric mean of all measurements.

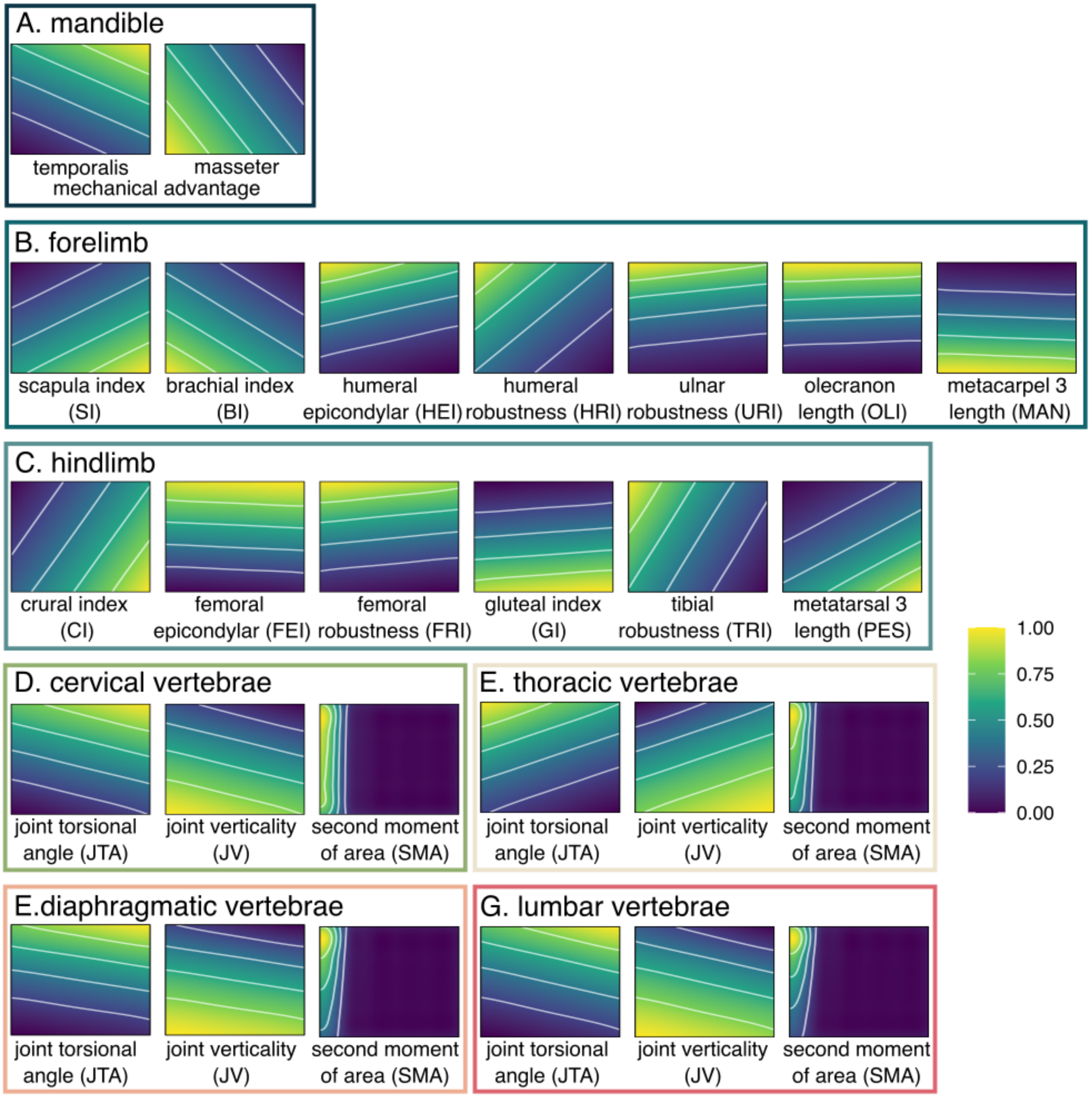

Fig. S7. Performance surfaces for each functional proxy based on non-size corrected morphological traits as part of Sensitivity Analyses 4, which investigated whether removing the effects of size on the morphological traits in creating the morphospace may potentially influence our performance surfaces and adaptive landscape results. The largest topological relief correlates with morphospace sample density, suggesting that the unevenness contributes to heterogeneous resolution affect the topology of the performance surfaces. Color represents height on the performance surface.

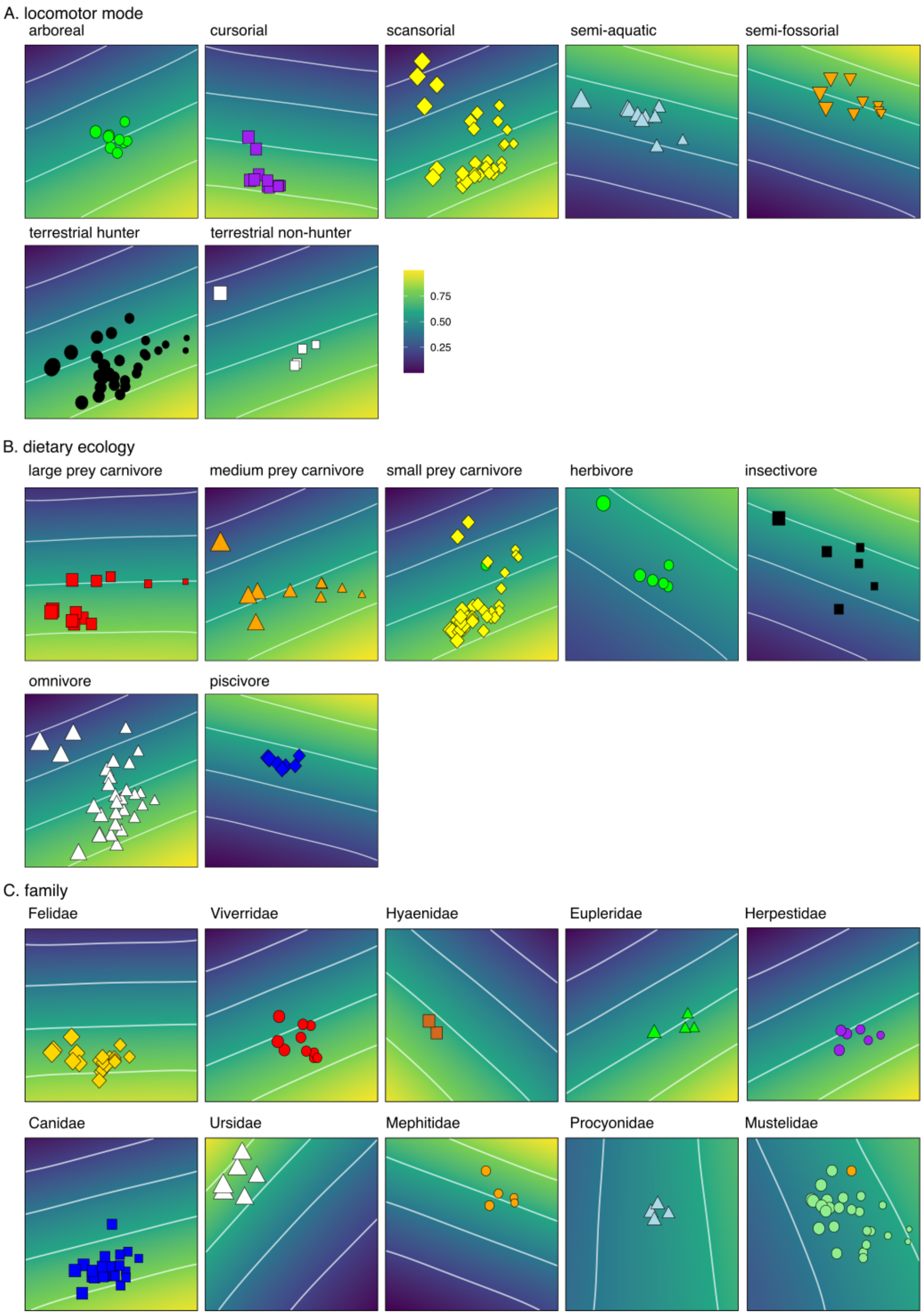

Fig. S8. Adaptive landscapes optimized for each locomotor, dietary, and familial groups as part of Sensitivity Analyses 4, which investigated whether removing the effects of size on the morphological traits in creating the morphospace may potentially influence our performance surfaces and adaptive landscape results. Using non-size corrected morphological traits does not change the topologies or weight partitions (Table S14–S11) of the landscapes when optimized for each locomotor group. See Tables S9–S11for breakdown of optima weights. See Figure 3 for original landscapes with all 27 functional proxies.

Table S14. Relative weights of each functional proxy optimized among the seven locomotor adaptive landscapes from Sensitivity Analyses 4, which investigated whether removing the effects of size on the morphological traits in creating the morphospace may potentially influence the optimized weights of the adaptive landscape. The values represent the mean weights of the traits calculated from the top 5% of landscapes.

functional proxies	arboreal	cursorial	scansorial	semi-aquatic	semi-fossorial	terrestrial hunter	terrestrial non-hunter
temMA	0.02	0.00	0.00	0.19	0.30	0.00	0.00
masMA	0.00	0.10	0.00	0.00	0.00	0.00	0.01
SI	0.09	0.05	0.18	0.00	0.00	0.21	0.13
BI	0.00	0.07	0.00	0.00	0.00	0.00	0.00
HRI	0.00	0.00	0.00	0.01	0.00	0.00	0.00
HEI	0.00	0.00	0.00	0.00	0.00	0.00	0.00
OLI	0.00	0.00	0.00	0.03	0.00	0.00	0.00
MAN	0.00	0.07	0.01	0.00	0.00	0.01	0.01
CI	0.02	0.00	0.01	0.00	0.00	0.00	0.01
FRI	0.00	0.00	0.00	0.02	0.00	0.00	0.00
GI	0.02	0.11	0.03	0.00	0.00	0.04	0.03
FEI	0.00	0.00	0.00	0.15	0.07	0.00	0.00
TRI	0.00	0.00	0.00	0.03	0.00	0.00	0.00
PES	0.05	0.02	0.07	0.00	0.00	0.09	0.07
URI	0.00	0.00	0.00	0.00	0.00	0.00	0.00
$SMA_{cervical}$	0.00	0.00	0.00	0.00	0.00	0.00	0.00
$JTA_{cervical}$	0.02	0.00	0.00	0.25	0.28	0.00	0.00
$JV_{cervical}$	0.00	0.13	0.01	0.00	0.00	0.01	0.01
$SMA_{thoracic}$	0.00	0.00	0.00	0.00	0.00	0.00	0.00
$JTA_{thoracic}$	0.00	0.00	0.00	0.00	0.00	0.00	0.00
$JV_{thoracic}$	0.73	0.16	0.62	0.05	0.00	0.57	0.63
SMA_{dia}	0.00	0.00	0.00	0.00	0.00	0.00	0.00
JTA_{dia}	0.00	0.00	0.00	0.10	0.10	0.00	0.00
JV_{dia}	0.02	0.16	0.03	0.00	0.00	0.04	0.07
SMA_{lumbar}	0.00	0.00	0.00	0.00	0.00	0.00	0.00
JTA_{lumbar}	0.00	0.00	0.00	0.16	0.25	0.00	0.00
JV_{lumbar}	0.02	0.15	0.03	0.00	0.00	0.03	0.03
Z-score	0.68	0.80	0.69	0.60	0.82	0.81	0.65

Table S15. Relative weights of each functional proxy optimized among the seven dietary adaptive landscapes from Sensitivity Analyses 4, which investigated whether removing the effects of size on the morphological traits in creating the morphospace may potentially influence the optimized weights of the adaptive landscape. The values represent the mean weights of the traits calculated from the top 5% of landscapes.

functional proxies	large prey carnivore	med prey carnivore	small prey carnivore	herbivore	insectivore	omnivore	piscivore
temMA	0.00	0.00	0.00	0.19	0.26	0.00	0.19
masMA	0.08	0.00	0.00	0.00	0.00	0.00	0.00
SI	0.04	0.15	0.22	0.06	0.02	0.17	0.00
BI	0.02	0.00	0.00	0.00	0.00	0.00	0.00
HRI	0.00	0.00	0.00	0.00	0.00	0.00	0.00
HEI	0.00	0.00	0.00	0.00	0.00	0.00	0.00
OLI	0.00	0.00	0.00	0.02	0.02	0.00	0.03
MAN	0.02	0.00	0.01	0.00	0.00	0.02	0.00
CI	0.00	0.02	0.00	0.02	0.01	0.02	0.00
FRI	0.00	0.00	0.00	0.01	0.01	0.00	0.03
GI	0.04	0.02	0.04	0.01	0.00	0.03	0.00
FEI	0.00	0.00	0.00	0.09	0.10	0.00	0.15
TRI	0.00	0.00	0.00	0.01	0.00	0.00	0.01
PES	0.01	0.08	0.09	0.03	0.01	0.08	0.00
URI	0.00	0.00	0.00	0.00	0.00	0.00	0.00
$SMA_{cervical}$	0.00	0.00	0.00	0.00	0.00	0.00	0.00
$JTA_{cervical}$	0.00	0.00	0.00	0.19	0.25	0.00	0.27
$JV_{cervical}$	0.06	0.02	0.01	0.00	0.00	0.02	0.00
$SMA_{thoracic}$	0.00	0.00	0.00	0.00	0.00	0.00	0.00
$JTA_{thoracic}$	0.00	0.00	0.00	0.00	0.00	0.00	0.00
$JV_{thoracic}$	0.43	0.65	0.57	0.16	0.06	0.63	0.01
SMA_{dia}	0.00	0.00	0.00	0.00	0.00	0.00	0.00
JTA_{dia}	0.00	0.00	0.00	0.08	0.10	0.00	0.12
JV_{dia}	0.19	0.04	0.03	0.01	0.00	0.03	0.00
SMA_{lumbar}	0.00	0.00	0.00	0.00	0.00	0.00	0.00
JTA_{lumbar}	0.00	0.00	0.00	0.12	0.17	0.00	0.17
JV_{lumbar}	0.11	0.02	0.01	0.00	0.00	0.02	0.00
Z-score	0.72	0.70	0.79	0.57	0.63	0.69	0.64

Table S16. Relative weights of each functional proxy optimized among the familial adaptive landscapes from Sensitivity Analyses 4, which investigated whether removing the effects of size on the morphological traits in creating the morphospace may potentially influence the optimized weights of the adaptive landscape. The values represent the mean weights of the traits calculated from the top 5% of landscapes.

functional proxies	Feli	Vive	Hyae	Eupl	Herp	Cani	Ursi	Meph	Proc	Must
temMA	0.00	0.00	0.00	0.02	0.00	0.00	0.00	0.41	0.23	0.21
masMA	0.02	0.00	0.66	0.00	0.00	0.00	0.00	0.00	0.00	0.00
SI	0.07	0.18	0.00	0.27	0.30	0.13	0.00	0.00	0.09	0.11
BI	0.02	0.00	0.02	0.00	0.00	0.01	0.00	0.00	0.00	0.00
HRI	0.00	0.00	0.00	0.00	0.00	0.00	0.23	0.00	0.00	0.00
HEI	0.00	0.00	0.00	0.00	0.00	0.00	0.02	0.00	0.00	0.00
OLI	0.00	0.00	0.00	0.00	0.00	0.00	0.02	0.00	0.00	0.00
MAN	0.04	0.00	0.00	0.00	0.00	0.03	0.00	0.00	0.00	0.00
CI	0.00	0.02	0.00	0.09	0.03	0.00	0.00	0.00	0.03	0.05
FRI	0.00	0.00	0.00	0.00	0.00	0.00	0.02	0.00	0.00	0.00
GI	0.09	0.03	0.02	0.00	0.02	0.08	0.00	0.00	0.00	0.01
FEI	0.00	0.00	0.00	0.00	0.00	0.00	0.03	0.03	0.03	0.04
TRI	0.00	0.00	0.02	0.00	0.00	0.00	0.57	0.00	0.00	0.00
PES	0.02	0.08	0.00	0.13	0.13	0.06	0.00	0.00	0.03	0.06
URI	0.00	0.00	0.00	0.00	0.00	0.00	0.01	0.00	0.00	0.00
$SMA_{cervical}$	0.00	0.00	0.00	0.00	0.00	0.00	0.00	0.00	0.00	0.00
$JTA_{cervical}$	0.00	0.00	0.00	0.00	0.00	0.00	0.02	0.30	0.14	0.16
$JV_{cervical}$	0.09	0.02	0.02	0.00	0.00	0.03	0.00	0.00	0.00	0.00
$SMA_{thoracic}$	0.00	0.00	0.00	0.00	0.00	0.00	0.00	0.00	0.00	0.00
$JTA_{thoracic}$	0.00	0.00	0.00	0.00	0.00	0.00	0.03	0.00	0.00	0.00
$JV_{thoracic}$	0.31	0.62	0.09	0.50	0.50	0.50	0.00	0.00	0.36	0.22
SMA_{dia}	0.00	0.00	0.00	0.00	0.00	0.00	0.00	0.00	0.00	0.00
JTA_{dia}	0.00	0.00	0.00	0.00	0.00	0.00	0.02	0.05	0.02	0.05
JV_{dia}	0.20	0.03	0.09	0.00	0.02	0.10	0.00	0.00	0.00	0.00
SMA_{lumbar}	0.00	0.00	0.00	0.00	0.00	0.00	0.00	0.00	0.00	0.00
JTA_{lumbar}	0.00	0.00	0.00	0.00	0.00	0.00	0.02	0.21	0.06	0.10
JV_{lumbar}	0.15	0.02	0.09	0.00	0.00	0.06	0.00	0.00	0.00	0.00
Z-score	0.81	0.77	0.69	0.73	0.78	0.85	0.88	0.85	0.62	0.62

Sensitivity Analyses 2: concern with number of partition weight increments

We conducted sensitivity analyses to investigate whether the coarse increments of partition weights may potentially influence the biological interpretation of our adaptive landscape results. We reduced the number of functional indices by using linear discriminant analyses (LDA) to determine which set of functional proxies best separated out the species among locomotor groups. We conducted one LDA for the limb dataset and a second LDA for the vertebral dataset (see Table S17 for linear discriminant functions). We used LD1 (accounts for 44% of the variance in the limb dataset and 54%% of the variance in the vertebral dataset) to determine the top functional proxies to retain. In the first analysis, we reduced the number of functional proxies to 14 by keeping both mandibular functional proxies (temMA and masMA), the top six vertebral functional proxies (JTA and JV of the cervical, thoracic, and diaphragmatic vertebrae), and the top six limb functional proxies (HRI, HEI, OLI, FRI, FEI, and TRI). This enabled us to reduce the weight partition steps to 0.15, creating a total of 77,520 possible weight combinations. In the second sensitivity analysis, we kept only the top three (JTA and JV of the cervical and JV of the thoracic) vertebral functional proxies and top three limb functional proxies (HRI, HEI, and FRI), enabling us to reduce the weight partition steps to 0.05, creating a total of 53,130 possible weight combinations. We then used the same procedure described in the Supplementary Methods to create performance surfaces and optimized adaptive landscapes. Both sensitivity analyses revealed similar landscape topologies and optimized weights (Fig. S9; Table S18) with the analyses using all 27 functional proxies (Fig. S3). This suggests that the number of functional proxies and subsequent number of steps has minimal impact in determining which traits are heavily weighted on the adaptive landscapes.

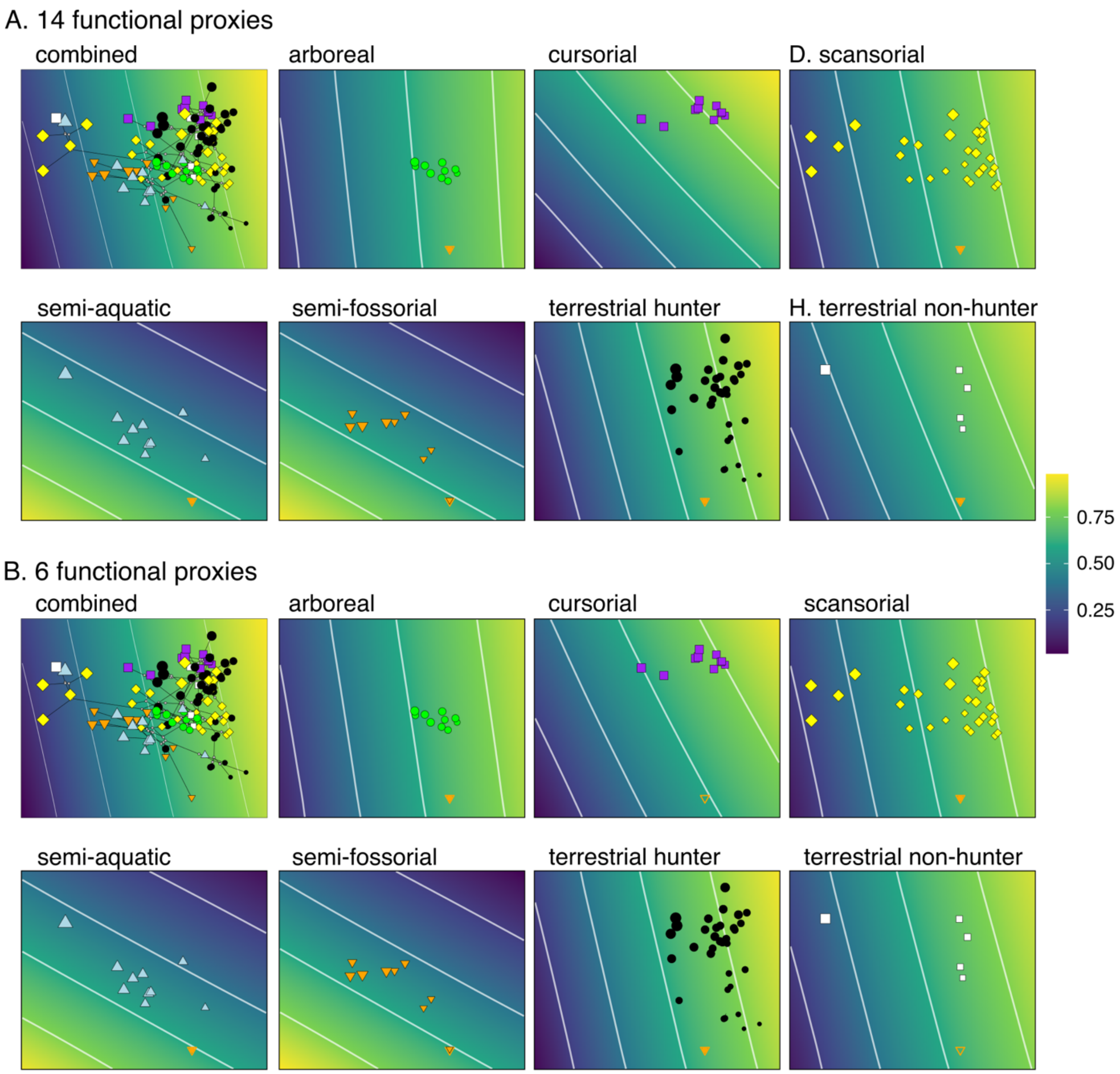

Fig. S9. Adaptive landscapes optimized for each locomotor group as part of Sensitivity Analyses 2, which investigated whether the coarse increments of partition weights may potentially influence our adaptive landscape results. Reducing the number of functional proxies from 27 to 14 (weight partition steps reduced to 0.15) or 6 (weight partition steps reduced to 0.07) does not change the topologies or weight partitions (Table S18) of the landscapes when optimized for each locomotor group. See Table S18 for breakdown of optima weights. See Figure 3 for original landscapes with all 27 functional proxies.

Table S17. Scaling matrix for the first linear discriminant function (LD1). Separate LDAs were performed for the limb and vertebral datasets.

functional proxies	LD1
limbs	
BI	3.307
CI	3.268
FEI	11.948
FRI	20.308
GI	0.690
HEI	33.182
HRI	-83.410
IM	0.474
MANUS	-0.506
OLI	10.067
PES	3.282
SI	-0.675
TRI	5.948
URI	-5.670
vertebrae	
$JTA_{cervical}$	-0.052
$JV_{cervical}$	0.052
Lateral $SMA_{cervical}$	0.000
Sagittal $SMA_{cervical}$	0.000
JTA_{lumbar}	-0.004
JV_{lumbar}	0.004
Lateral SMA_{lumbar}	0.000
Sagittal SMA_{lumbar}	0.000
$JTA_{thoracic}$	-0.013
$JV_{thoracic}$	0.013
Lateral $SMA_{thoracic}$	0.000
Sagittal $SMA_{thoracic}$	0.000
JTA_{dia}	-0.013
JV_{dia}	0.013
Lateral SMA_{dia}	0.000
Sagittal SMA_{dia}	0.000

Table S18. Relative weights of each functional proxy optimized among the seven locomotor adaptive landscapes from Sensitivity Analyses 2, which investigated whether the coarse increments of partition weights may potentially influence our adaptive landscape results. (**A**) Relative weights of proxies when the number of proxies was reduced to 14 and incremental weight partitions was reduced to 0.15. (**B**) Relative weights of proxies when the number of proxies was reduced to 6 and incremental weight partitions was reduced to 0.07. The values represent the mean weights of the traits calculated from the top 5% of landscapes.

functional proxies	arboreal	cursorial	scansorial	semi-aquatic	semi-fossorial	terrestrial hunter	terrestrial non-hunter
A. number of proxies reduced to 14							
temMA	0.01	0.00	0.01	0.03	0.04	0.00	0.00
masMA	0.04	0.00	0.02	0.01	0.01	0.03	0.01
HRI	0.00	0.00	0.00	0.01	0.00	0.00	0.01
HEI	0.00	0.00	0.00	0.01	0.01	0.00	0.00
OLI	0.01	0.00	0.01	0.05	0.06	0.00	0.00
FRI	0.01	0.00	0.00	0.03	0.03	0.00	0.00
FEI	0.04	0.00	0.02	0.43	0.42	0.00	0.02
TRI	0.00	0.00	0.00	0.01	0.00	0.00	0.01
$JTA_{cervical}$	0.04	0.00	0.02	0.35	0.36	0.00	0.01
$JV_{cervical}$	0.01	0.33	0.02	0.00	0.00	0.03	0.06
$JTA_{thoracic}$	0.00	0.00	0.00	0.01	0.01	0.00	0.00
$JV_{thoracic}$	0.79	0.33	0.80	0.02	0.01	0.86	0.64
JTA_{dia}	0.01	0.00	0.01	0.06	0.06	0.00	0.01
JV_{dia}	0.04	0.33	0.10	0.00	0.00	0.09	0.21
Z	0.69	0.82	0.69	0.69	0.75	0.83	0.64
B. number of proxies reduced to 6							
HRI	0.01	0.01	0.01	0.03	0.02	0.01	0.02
HEI	0.01	0.01	0.01	0.04	0.04	0.01	0.02
FRI	0.02	0.01	0.02	0.07	0.07	0.01	0.02
$JTA_{cervical}$	0.06	0.01	0.04	0.81	0.84	0.02	0.03
$JV_{cervical}$	0.03	0.47	0.06	0.01	0.01	0.05	0.09
$JV_{thoracic}$	0.87	0.49	0.87	0.05	0.03	0.91	0.82
Z	0.69	0.79	0.69	0.68	0.74	0.82	0.64

References

Boszczyk BM, Boszczyk AA, Putz R. 2001. Comparative and functional anatomy of the mammalian lumbar spine. Anatomical Rec 264:157–68.

Christiansen P. 2002. Locomotion in terrestrial mammals: the influence of body mass, limb length and bone proportions on speed. Zoöl J Linn Soc 136:685–714.

Davis DD. 1964. The giant panda: a morphological study of evolutionary mechanisms Fieldiana (Zoology Memoirs).

Dickson BV, Clack JA, Smithson TR, Pierce SE. 2021. Functional adaptive landscapes predict terrestrial capacity at the origin of limbs. Nature 589:242–45.

Dickson BV, Pierce SE. 2019. Functional performance of turtle humerus shape across an ecological adaptive landscape. Evolution 73:1265–77.

Fischer MS, Schilling N, Schmidt M, Haarhaus D, Witte H. 2002. Basic limb kinematics of small therian mammals. J Exp Biol 205:1315–38.

Garland T, Janis CM. 1993. Does metatarsal/femur ratio predict maximal running speed in cursorial mammals? J Zoöl 229:133–51.

Gasc J-P. 2001. Comparative aspects of gait, scaling and mechanics in mammals. Comp Biochem Physiol Part A: Mol Integr Physiol 131:121–33.

Greaves WS. 2012. The Mammalian Jaw. 3–25.

Hildebrand M. 1985. Digging of Quadrupeds. In: Hildebrand M, Bramble DM, Liem KF, Wake DB, editors. Functional Vertebrate Morphology Cambridge, MA: Harvard University Press. p. 89–109.

Jones KE, Dickson BV, Angielczyk KD, Pierce SE. 2021. Adaptive landscapes challenge the "lateral-to-sagittal" paradigm for mammalian vertebral evolution. Curr Biol 31:1883-1892.e7.

Jones KE, Gonzalez S, Angielczyk KD, Pierce SE. 2020. Regionalization of the axial skeleton predates functional adaptation in the forerunners of mammals. Nat Ecol Evol 4:470–78.

Kemp TJ, Bachus KN, Nairn JA, Carrier DR. 2005. Functional trade-offs in the limb bones of dogs selected for running versus fighting. J Exp Biol 208:3475–82.

Kilbourne BM. 2017. Selective regimes and functional anatomy in the mustelid forelimb: Diversification toward specializations for climbing, digging, and swimming. Ecol Evol 66:2369–12.

Kilbourne BM, Hoffman LC. 2013. Scale Effects between Body Size and Limb Design in Quadrupedal Mammals. PLoS ONE 8:e78392.

Kram R, Taylor CR. 1990. Energetics of running: a new perspective. Nature 346:265–67.

Lagaria A, Youlatos D. 2006. ANATOMICAL CORRELATES TO SCRATCH DIGGING IN THE FORELIMB OF EUROPEAN GROUND SQUIRRELS (SPERMOPHILUS CITELLUS). J Mammal 87:563–70.

Law CJ. 2019. Solitary meat-eaters: solitary, carnivorous carnivorans exhibit the highest degree of sexual size dimorphism. Sci Rep-uk 9:15344.

Law CJ. 2021. Ecological drivers of carnivoran body shape evolution. Am Nat 198:406–20.

Law CJ, Blackwell EA, Curtis AA, Dickinson E, Hartstone-Rose A, Santana SE. 2022. Decoupled evolution of the cranium and mandible in carnivoran mammals. Evolution 76:2959–74.

Law CJ, Young C, Mehta RS. 2016. Ontogenetic scaling of theoretical bite force in southern sea otters (Enhydra lutris nereis). Physiol Biochem Zool 89:347–63.

Lessa EP, Stein BR. 1992. Morphological constraints in the digging apparatus of pocket gophers (Mammalia: Geomyidae). Biol J Linn Soc 47:439–53.

Martín-Serra A, Pérez-Ramos A, Pastor FJ, Velasco D, Figueirido B. 2021. Phenotypic integration in the carnivoran backbone and the evolution of functional differentiation in metameric structures. Evolution Letters 1–14.

Polly PD. 2007. Limbs in mammalian evolution. In: Hall BK, editor. Fins into Limbs Evolution, Development, and Transformation. Fins into Limbs Evolution, Development, and Transformation Fins into Limbs Evolution, Development, and Transformation. p. 245–68.

Polly PD, Lawing AM, Fabre A-C, Goswami A. 2013. Phylogenetic principal components analysis and geometric morphometrics. Hystrix, the Italian Journal of Mammalogy 24:33–41.

Polly PD, Stayton CT, Dumont ER, Pierce SE, Rayfield EJ, Angielczyk KD. 2016. Combining Geometric Morphometrics and Finite Element Analysis with Evolutionary Modeling: Towards a Synthesis. J Vertebr Paleontol 36:e1111225.

Pontzer H. 2007a. Effective limb length and the scaling of locomotor cost in terrestrial animals. J Exp Biol 210:1752–61.

Pontzer H. 2007b. Predicting the energy cost of terrestrial locomotion: a test of the LiMb model in humans and quadrupeds. J Exp Biol 210:484–94.

Rickman J, Burtner AE, Linden TJ, Santana SE, Law CJ. 2023. Size And Locomotor Ecology Have Differing Effects on the External and Internal Morphologies of Squirrel (Rodentia: Sciuridae) Limb Bones. Integr Org Biol 5:obad017.

Rose J, Moore A, Russell A, Butcher M. 2014. Functional osteology of the forelimb digging apparatus of badgers. J Mammal 95:543–58.

Samuels JX, Meachen JA, Sakai SA. 2013. Postcranial morphology and the locomotor habits of living and extinct carnivorans. J Morpho 274:121–46.

Samuels JX, Valkenburgh BV. 2008. Skeletal indicators of locomotor adaptations in living and extinct rodents. J Morpho 269:1387–1411.

Santana SE, Grosse IR, Dumont ER. 2012. Dietary hardness, loading behavior, and the evolution of skull form in bats. Evolution 66:2587–98.

Schmidt M, Fischer MS. 2009. Morphological integration in mammalian limb proportions: dissociation between function and development. Evolution 63:749–66.

Smith JM, Savage RJG. 1956. Some locomotory adaptations in mammals. J Linn Soc Lond Zoology 42:603–22.

Strang KT, Steudel K. 1990. Explaining the scaling of transport costs: the role of stride frequency and stride length. J Zoöl 221:343–58.

Throckmorton GS, Dean JS. 1994. The relationship between jaw-muscle mechanical advantage and activity levels during isometric bites in humans. Arch Oral Biol 39:429–37.
